# Computational and experimental evaluation of *Pisolithus arhizus* metabolites targeting major efflux pumps of mastitis-associated *Staphylococcus aureus*

**DOI:** 10.1371/journal.pone.0354013

**Published:** 2026-07-16

**Authors:** Hansa Gul, Iram Zahra, Zahida Nasreen, Haris Ahmed Khan, Muhammad Naeem-ul-Hassan, Nouman Ahmad, Nasir Assad, Dawit Kifle, Lennin Garrido-Palazuelos

**Affiliations:** 1 Department of Zoology, University of Mianwali, Mianwali, Pakistan; 2 Institute of Chemistry, University of Sargodha, Sargodha, Pakistan; 3 Department of Biotechnology, University of Mianwali, Mianwali, Pakistan; 4 College of Natural Sciences, Department of Biology, Jimma University, Jimma, Ethiopia; 5 College of Natural and Computational Sciences, Department of Biology, Mizan-Teppi University, Teppi, Ethiopia; 6 Departamento Académico de Ciencias de la Salud, Universidad Autónoma de Occidente, Los Mochis, Sinaloa, Mexico; University of Westminster - Regent Street Campus: University of Westminster, UNITED KINGDOM OF GREAT BRITAIN AND NORTHERN IRELAND

## Abstract

Antimicrobial resistance (AMR) in pathogenic bacteria, particularly *Staphylococcus aureus*, is an increasing global concern in veterinary medicine. The present study evaluated bioactive metabolites from *P. arhizus* as potential inhibitors of major *S. aureus* efflux pumps. The methanolic extracts of *P. arhizus* were profiled using GC–MS to identify the main constituents, and their antibacterial activity against *S. aureus* was evaluated using a well diffusion assay. The experimental results showed that the crude methanolic extract exhibited a 20 mm zone of inhibition with a MIC of 30 µg/mL. Furthermore, isolated metabolites, octadecanoic acid and compound tentatively identified by GC-MS as 3-(6-methyl-3-pyridyl)-1,5-diphenyl-2-pyrazoline (a pyrazoline derivative), (tentatively identified) demonstrated zones of inhibition of 19 mm each, with MIC values of 30 µg/mL and 40 µg/mL, respectively**.** *In silico* analyses, including molecular docking and molecular dynamics (MD) simulations, were performed to examine the binding and stability of 12 fungal metabolites with the major *S. aureus* efflux pumps NorA, NorB, NorC, and MepA. Binding-free energy estimation by MM-GBSA supported favorable interactions for selected compounds, with pyrazoline and octadecanoic acid showing the most promising profiles. Principal component analysis (PCA), dynamic cross-correlation matrix (DCCM) analysis, and ADMET predictions further suggested stable complex behavior and acceptable drug-likeness features. In general, these results indicate that the *P. arhizus* metabolites demonstrated antibacterial activity and showed a high binding affinity to efflux pumps *in silico*, suggesting that they merit further investigation as potential antibacterial agents with predicted interactions toward efflux pump proteins.

## 1. Introduction

Dairy farming is an important sector that supports the global milk supply and human nutrition [1–3]. Maintaining optimal udder health is essential to ensure milk quality and preserve the integrity of the dairy production chain [4,5]. Among the major health challenges affecting dairy herds, mastitis remains one of the most prevalent and economically burdensome diseases worldwide [6].

Mastitis is a complex and multifaceted inflammatory medical condition of the mammary glands (MG) that is mainly linked to bacterial infections and caused by over 200 pathogenic microorganisms. The disease causes a decrease in milk yield and composition and elevates somatic cell counts, which may make milk unfit for consumption [7]. *Staphylococcus aureus* is a common etiological pathogen among other bacterial agents that cause mastitis. It is known to possess several determinants of virulence, such as toxin production, biofilm formation, and antimicrobial resistance (AMR) [8]. Mastitis has a significant economic effect; each case of mastitis has been estimated to cost USD 80–125 per cow as a result of reduced productivity, increased veterinary treatment, and culling. Mastitis is reported to be among the most common livestock diseases in several regions, such as Pakistan, making it a highly relevant disease on a global scale [9].

Defense of the udder depends on a coordinated adaptive and innate immune response. The presence of keratin in the teat canal is also a major barrier as it traps pathogens and helps in the disruption of membranes. Furthermore, antimicrobial factors such as lysozyme, lactoferrin, and lactoperoxidase reduce bacterial growth, and immune cells such as neutrophils, macrophages, and dendritic cells mediate cytokine production and pathogen clearance [10]. Despite these defenses, *S. aureus* acts as an opportunistic pathogen capable of colonizing the teat canal and invading mammary epithelial cells, causing chronic intramammary infections (IMI) that are challenging to eliminate [11,12]. Effective persistence is enabled by the presence of various virulence factors that aid in adhesion, invasion, and immunocompromising, most of which are anchored by the genome and/or linked to infectious genetic factors [13].

In addition, the widespread use of antibiotics in dairy product production has led to the rise and spread of multidrug resistant strains (MDR) strains. Efflux pumps NorA, NorB, NorC, and MepA play an important role in the development of AMR in *S. aureus*. These membrane-bound transporters decrease the concentration of antibiotics inside cells through the active excretion of antimicrobial agents and other toxic substances, thereby lowering the effect of drugs. Efflux pumps also have the ability to cause cross-resistance through the export of structurally unrelated compounds, which complicates treatment strategies and is correlated with treatment failure in veterinary and human settings [14]. NorA is a member of the major facilitator superfamily (MFS) and is specifically linked to the extrusion of fluoroquinolones, biocides, and various naturally occurring molecules [15–17]. Other efflux pumps, including NorB, NorC, and MepA, of the MFS and MATE, are also essential for the multidrug-resistant phenotype of *S. aureus* [18,19]. Although NorA has been studied in depth, these secondary pumps act as secondary protection for bacteria, allowing them to survive in the presence of NorA. Consequently, the ability to identify natural metabolites that simultaneously inhibit several efflux systems is a stronger approach to overcome bacterial resistance in bovine mastitis.

Owing to the increasing burden of antibiotic resistance, the identification of new complementary or alternative antimicrobial agents is a priority. *Pisolithus arhizus* is a basidiomycete sclerodermataceae fungus that represents the ecological diversity and bioactivity of fungal metabolites [20]. Pisolithus species are found in various ecosystems on six continents and have distinctive fruiting bodies (basidiocarps). *P. arhizus* is highly tolerant to the environment and grows in acidic geothermal soils and mining waste at extreme pH and temperature environments. It is also a mutually beneficial association with economically important trees, including *Quercus* and *Eucalyptus*, which improves forest productivity and ecosystem resilience [21,22]. Mycochemical studies have identified pulvinic acid derivatives and pisoquinone as the main contributors to its characteristic red-brown pigmentation [23], along with triterpenes [24]. In addition, antifungal benzoic acid derivatives, including pisolithin A and B, have been isolated from the liquid cultures.

In the present study, gas chromatography mass spectrometry (GC–MS) was used to profile *P. arhizus* methanolic extracts and identify the major metabolites. An *in silico* workflow was implemented to assess the potential of the identified compounds as antibacterial candidates for mastitis, with the major efflux pumps NorA, NorB, NorC, and MepA of *Staphylococcus aureus* as molecular targets. ADME analysis was used to evaluate the pharmacokinetic and drug-like properties. Molecular docking was used to determine the binding affinity and binding modes, and molecular dynamics (MD) studies were conducted to determine the stability and conformational complexes between the ligand and proteins over time. Furthermore, MM-GBSA was used to estimate the binding free energies. These analyses aimed to provide an understanding of fungal metabolites that can be used as potential efflux pump inhibitor candidates and adjuncts to antibiotics in mastitis-related *S. aureus.*

## 2. Materials and methods

**Fig 1:**
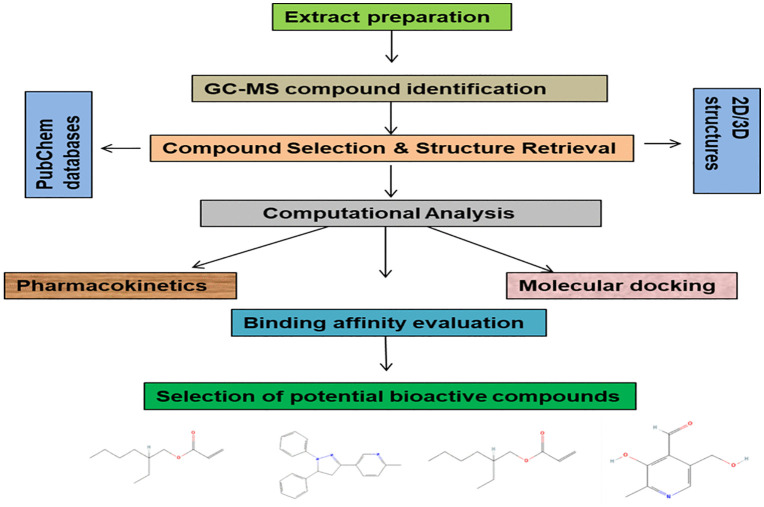
Methodological flow chart of the study.

In this study, *P. arhizus* was characterized using GC–MS, FT-IR, and computational methods. Fig 1 summarizes the analysis workflow.

### 2.1 Collection and preparation

The fungus *P. arhizus* was collected from Delay Shankai Bromikhail, Tehsil Sararogha, South Waziristan, Khyber Pakhtunkhwa (KPK), Pakistan (32.3 ° N, 69.8 ° E). The sample was obtained from privately owned land belonging to the authors; therefore, no specific collection permits were required. All procedures were performed in accordance with the relevant national and international guidelines. Taxonomic identification was performed at the Department of Botany, University of Sargodha (Punjab, Pakistan). The collected material was placed in a zipper bag (voucher no. SARGU/CHEM-098), transported to the Institute of Chemistry, University of Sargodha, and air-dried at room temperature for 20 days. The dried samples were ground into a fine powder using an electric grinder. For extraction, 5 g of powdered fungal material was mixed with 50 mL of 80% (v/v) methanol (methanol:water, 80:20) in a 1000 mL volumetric flask and processed as described by Assad et al. (2025) [25] with minor modifications.

The suspension was stirred magnetically on a hot plate (25 °C) for 3 h to facilitate extraction. The mixture was filtered through a Whatman No. 42 filter paper. The filtrate was dried in an air-drying oven at 45 °C for 24 h, and the dried extract was stored for further analysis. The crude extract was reconstituted in sterile dimethyl sulfoxide (DMSO) at a stock concentration of 20 mg/ ml for bioassays. For the well-diffusion assay, working solutions were prepared from the stock solution diluted in sterile water, ensuring that the final concentration of DMSO did not exceed 1% (v/v) to prevent any antibacterial effects caused by the solvent.

### 2.2 Fourier transform infrared (FTIR) spectrometry analysis

The functional groups of the samples were identified using FTIR spectroscopy. Spectral data were recorded in KBr pellets using a Shimadzu FTIR 8400S spectrometer over the range of 4000–500 cm ^−1^.

### 2.3 Gas chromatography-mass spectrometry (GC–MS) analysis

The methanolic extract of *P. arhizus* was prepared by dissolving dried fungal powder in 80% methanol, followed by filtration and concentration under reduced pressure. GC–MS analysis was performed on the concentrated extract to identify volatile and semi-volatile metabolites, including fatty acids, phthalates, xylene derivatives, and pyrazoline derivatives. The extract was sufficiently volatile for detection without any chemical derivatization. Measures were taken to avoid contamination, and previous studies have successfully profiled similar extracts using GC-MS. [26–28].

#### 2.3.1 The GC-MS conditions.

The bioactive ingredients in *P. arhizus* were isolated and quantitatively studied following refinement of the approach described by Nasir Shah et al. (2023) [29]. GC–MS analysis was performed using an Agilent 5977B system, and the total run time was 36 min. The process began with an oven temperature of 70 °C, which was maintained for 3 min. Then, there was a 10-degree ramp every min until the temperature reached 270 °C, which was maintained for 13 min. An infusion volume of 1 µL was supplied by the ALS injection source and a 10 µL injector was used in split mode with a split ratio. A steady flow rate of 1 ml/ min was maintained for the carrier gas, helium. The column, which had a maximum working temperature of 325 °C, was a DB-1 (30 m × 0.25 mm × 0.25 µm). At 280 °C, the MSD transfer line was kept constant. The mass spectrometer was used in the scan mode, covering a mass range of 30–650 amu, with a solvent delay of 2 min. The temperature of the ion source was 230 °C, while the temperature of the quadrupole was 150 ° C. The standard scanning mode was used to set the electromotive force (EM) voltage to a gain factor of 1.0. In this procedure, neither trace-ion detection nor timed events were used.

### 2.4 Antibacterial evaluations by well diffusion method

All antibacterial tests were performed on *Staphylococcus aureus* (ATCC 23235). This strain of methicillin-sensitive *staphylococcus aureus* (MSSA) has a well-studied susceptibility profile and was obtained from the American Type Culture Collection (ATCC, Manassas, VA, USA) [28]. The bacteria were grown on Mueller-Hinton Agar (MHA) plates at 37 °C, and mid-log phase cultures were used to prepare the bacterial lawns for both the well diffusion assay and test tubes containing the MIC) assay. The test compounds, including the pyrazoline derivative (pyrazoline, CAS: 628595-21-3) and octadecanoic acid (CAS: 57-11-4), were obtained in pure form from Sigma-Aldrich (St. Louis, MO, USA) and used without further purification. Stock solutions (10 mg/ ml) in DMSO) were stored at 4 °C, and working solutions were freshly prepared with a final concentration of DMSO <1% v/v to avoid antibacterial effects from the solvent.

The antibacterial potential of the *P. arhizus* extract and its compounds was evaluated using the well diffusion method, following the protocols of Assad et al. (2024) and Gul et al. (2025), with a few modifications [30,31]. Wells with a diameter of 6 mm were created in the MHA plates, and in well A, 20 µg/mL tetracycline, in well B, extract of *P. arhizus,* in well C, 20 µg/mL pyrazolone, and in well D, 20 µg/mL octadecanoic acid were added. Tetracycline (20 µg/mL) served as a positive control for efflux inhibition, while wells containing only DMSO acted as negative controls. Plates were incubated at 37 ° C for 24 h, and the zones of inhibition around each well were measured in millimeters (mm) using a digital caliper.

A serial dilution assay was conducted to identify the MIC using 10, 20, 30, 40, 50, and 60 µg/mL of each test compound and the *P. arhizus* extract, with slight modifications adopted by Gul et al. (2026) [32]. The dilutions were made in sterile MH broth and inoculated in sterile test tubes containing approximately 5 × 10^5^ CFU/mL) of *S. aureus,* per tube. The incubation of The tubes at 37 ° C was followed by a 24 h period of incubation, after which a visual assessment of the bacterial growth was performed. The MIC was determined as the lowest concentration of the compound or extract that prevented visible bacterial growth.

#### 2.4.1 Statistical analysis.

All experiments were performed in triplicate, and the results are presented as mean standard deviation (SD). Statistical significance between groups was determined using one-way analysis of variance (ANOVA) followed by Tukey’s HSD post-hoc test, with p _< 0.05_ considered statistically significant. Analyses were performed using Statistix 8.1 software.

### 2.5 Molecular docking

#### 2.5.1 Retrieval and preparation of ligands.

GC-MS analysis revealed 14 high-intensity peaks representing 12 different chemical structures, some of which were identified as putative isomers of the active compounds. Tetracycline was used as a reference antibiotic to facilitate the comparative evaluation of the physicochemical and pharmacological characteristics of fungal metabolites. Three-dimensional molecular structures (in SDF format) were extracted using BIOVIA and converted to the protein data bank (PDB) format using BIOVIA Discovery Studio v2021 (S1 Table). Ligand preparation was performed using AutoDockTools (ADT), which included the addition of polar hydrogen atoms, assignment of Gasteiger charges, and definition of rotatable bonds. The prepared ligands were subsequently saved in PDBQT format for docking calculations [33,34]. This preparation step ensured standardized ligand input files for downstream docking and simulation analysis.

#### 2.5.2 *In silico* ADMET and drug-likeness profiling of fungal metabolites.

The pharmacokinetic and toxicological behaviors of the identified myco-compounds were predicted using two online web servers, SwissADME (http://www.swissadme.ch) [35] and pkCSM (https://biosig.lab.uq.edu.au/pkcsm/prediction). These tools were used to evaluate the suitability of each compound as a potential drug candidate by submitting the canonical SMILES and sdf files of each compound [36–39].

Computational predictions of physicochemical, pharmacokinetic, and medicinal chemistry properties were performed to evaluate the drug-like potential of the myco-compounds. In particular, it included important physicochemical descriptors (e.g., molecular weight and lipophilicity), pharmacokinetic properties (e.g., gastrointestinal absorption, blood-brain barrier permeability, and skin permeability), and the critical medicinal chemistry metrics of P-glycoprotein substrate docking affinity, toxicity potential, drug similarity, and bioavailability score [35].

Drug-likeness is primarily evaluated using empirical rules and filters. Lipinski’s rule of five was applied with the following criteria: molecular weight ≤ 500 g/mol, calculated octanol/water partition coefficient (cLogP) ≤ 5, hydrogen-bond donors ≤ 5, and hydrogen-bond acceptors ≤ 10. Compounds with more than one violation were considered less likely to exhibit favorable oral bioavailability [40,41]. In addition, Veber’s criteria were assessed, including a topological polar surface area (TPSA) of ≤ 140 Å^2^ and ≤ 10 rotatable bonds, which are commonly associated with improved oral bioavailability. Aqueous solubility was estimated using the ESOL model (logS), which yields an empirical approximation of solubility based on molecular descriptors. Together, these *in silico* parameters were used to rank metabolites with desirable predicted pharmacokinetic properties and acceptable safety profiles for subsequent structure-based investigations.

#### 2.5.3 Selection of target protein.

Molecular docking was conducted with *S. aureus* efflux transporters as target receptors (NorA, NorB, and NorC and MepA) For the structure of each candidate compounds, we used its crystallized complex from pubchem database. Since experimentally resolved structures were not available for all targets, a combination of predicted and experimentally determined models was used. Specifically, the three-dimensional structures of NorA (AlphaFold entry AF-Q53459-F1, strain N315), NorB (AF-Q2FH03-F1), and MepA (AF-Q2YVH4-F1) were obtained from the AlphaFold protein structure database (https://alphafold.ebi.ac.uk/), and the NorC structure was recovered from the Protein Data Bank (PDB ID: 7D5P). All AlphaFold models displayed high confidence (average pLDDT > 85). The preparation was performed using AutoDockTools (ADT). Polar hydrogen atoms were added, nonprotein atoms or molecules (when present) were removed, and Kollman partial charges were assigned [42]. The receptor structure was then saved in the PDBQT format for use in docking calculations [43].

#### 2.5.4 Molecular docking.

Drug-likeness filters (such as Lipinski’s rule of five) were used to rank the metabolites to be screened by structure, and the results of the molecular docking assay (with the selected efflux transporters, namely NorA, NorB, NorC, and MepA) were tested in the AlphaFold Protein Structure Database and the PDB (S1 Fig). Docking calculations were performed using the PyRx v0.8 code, which uses the AutoDock Vina engine [44]. The receptor and ligands were combined in the form of prepared receptor and ligands (PDBQT format) imported into PyRx, and the docking scores were reported as predicted binding affinities (kcal/mol) [45]. The top-ranked pose (i.e., the conformation with the lowest predicted binding energy) of each ligand was saved for further interaction analysis (including key interacting residues and interaction types). The RMSD values reported by AutoDock Vina were used to compare alternative poses relative to the best-ranked conformation and to support pose consistency. Docking was performed according to the grid parameters listed in Table 1.

**Table 1:** Grid Parameters for docking of targeted proteins.

Box Size X (Å)	25x25x25		
Protein	Grid Center X (Å)	Grid Center Y (Å)	Grid Center Z (Å)
NorA	−1.9814	−1.6752	2.5954
NorB	1.5812	0.6305	0.0738
NorC	−2.1243	27.5796	−57.5137
MepA	−1.0178	−0.0821	0.4270

### 2.6 Molecular dynamics simulation

Molecular dynamics (MD) simulations were performed using GROMACS v2018 to evaluate the structural stability and interaction dynamics of the selected proteins in complex with the top-ranked ligands identified by molecular docking. Given the absence of an experimentally resolved structure, the NorA receptor model was obtained from the AlphaFold Protein Structure Database (AlphaFold entry AF-P0A0J5-F1; S1 Fig). Each protein–ligand complex was parameterized using a force field (40) and solvated in a triclinic simulation box with TIP3P water. The solute was centered in the box, and a minimum distance of 1.0 nm was maintained between the complex and the box edges. The system was neutralized by adding counter ions (Na⁺ and/or Cl^−^) as required. Energy minimization was performed using the steepest descent algorithm for up to 50,000 steps. The minimized systems were then equilibrated for a total of 100 ps under NVT and NPT conditions prior to the production runs. Production MD simulations were performed for 50 ns for each Nor(A,B,C, and MepA)–ligand complex. The trajectory was analyzed to calculate structural descriptors, including the mean square deviation (RMSD), root-mean-square fluctuation (RMSF), radius of gyration (Rg), and potential energy. Plots were generated using XMGrace [43].

### 2.7 MM-GBSA-based binding-free energy calculation

The g_mmpbsa tool was used to estimate the binding free energies of the Nor (A, B, and MepA) ligand complexes from molecular dynamics (MD) trajectories. This approach applies the molecular mechanics/generalized Born surface area (MM/GBSA) method to approximate the binding free energy by combining molecular mechanics energy terms with implicit-solvent contributions. Briefly, the total binding free energy (ΔG_bind) was calculated as the difference between the free energies of the complex and its separated components (protein and ligand), providing an efficient estimate of the thermodynamic favorability of ligand binding and supporting the comparative ranking of the docked candidates. [46].

The ΔG_bind_ energy was estimated as the sum of the van der Waals energy (ΔE_vdw_) and electrostatic energy (ΔE_ele_). The total binding free energy was calculated using the following equation (Eq 1):


$$\begin{array}{c} \Delta \text{Gbind}=\Delta \text{EvdW}+\Delta \text{Eele}+\Delta \text{Gpolar}+\;\Delta \text{Gnonpolar}-\text{T}\Delta \text{S} \end{array}$$ (1)

ΔEvdw = van der Waals interaction

ΔEele = electrostatic interactions

−TΔS = (Entropy contribution)

This method provides a more accurate and reliable estimate of ligand-binding affinity than conventional scoring functions.

### 2.8 Data interpretation

A well diffusion assay was used to measure antibacterial activity and the diameter of the inhibition zone was measured in mm as an indicator of growth inhibition. For replicate measurements, the results were summarized with descriptive statistics (e.g., mean standard deviation) to determine measurement consistency over repeats. The results of the computational methodologies of molecular docking, MD simulations, and MM-GBSA calculations were compared to determine the extent of the ligand-binding propensity and complex stability to the NorA efflux transporter. A preliminary ranking of the ligands in terms of predicted binding affinity was performed using docking scores. Standard stability measures (such as root-mean-square deviation [RMSD], root-mean-square fluctuation [RMSF], radius of gyration [Rg], and potential energy) were analyzed, and the stability of the MD trajectories was determined by convergence behavior (e.g., plateauing of RMSD) and by the fact that the MD trajectory avoids any large-scale deviation. The binding free energies of the MM-GBSA were interpreted in comparison to enable the prioritization of ligands with relatively consistent favorable energetic profiles. Since the *in silico* workflow was exploratory and the experimental assay was primarily screening based, inferential statistical hypothesis testing was not implemented and rather reproducibility was facilitated by the reproducibility metrics of replicate consistency (where available) and convergence/stability based on MD trajectories.

## 3. Results

### 3.1 Fourier transform infrared (FTIR) spectrometry analysis

The FTIR spectrum of the methanolic extract revealed the presence of several functional groups associated with bioactive compounds. Comparison with previous studies revealed both similarities and differences in the detected functional groups. The most significant bands are the OH band (3610 cm^-1^), C = O band (1741 cm^-1^), and NH bend at 1541 cm^-1^. The results obtained are consistent with those of recent studies, especially those on the antifungal and functional specifications of *Tamarix aphylla* extracts. The analysis revealed strong O-H and C = O bands, indicating the presence of phenolic and carbonyl components [47].

**Fig 2:**
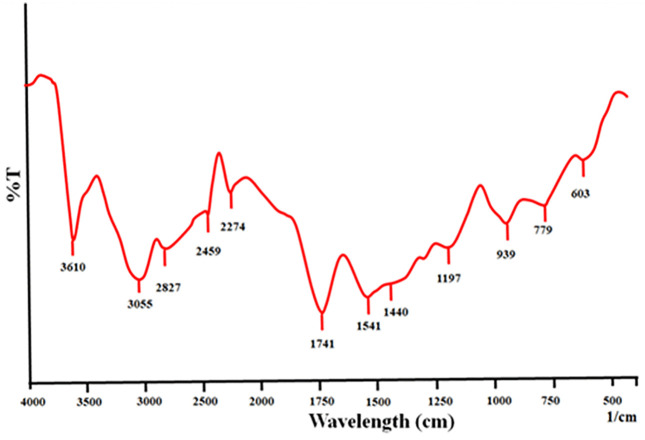
FTIR spectrum of the methanolic extract of *P. arhizus.*

The spectrum shows significant absorption bands of stretch hydroxyl (OH) at 3610 cm^-1^, alkene (C-H), carbonyl (CH = O), and ether/ester (C-O) at 1741 and 1197 cm-1, respectively, indicating the presence of bioactive compounds with antimicrobial potential [31] (Fig 2).

The (C = O) and (O-H) hydroxyl groups are vital for generating antimicrobial effects. These properties are strongly associated, mainly because of the presence of fatty acids and phenolic compounds. These results are analogous to our inferences and support the theory that the chemical composition of fungi consists of substances that exhibit important biological properties (Table 2).

**Table 2:** Functional groups present in the methanolic extract of *P. arhizus* fungus*.*

Wavelength (cm^−^^1^)	Functional Group	Bond Type
3610	O-H Stretch	Hydroxyl Group
3055	C-H Stretch	Alkene
2827	C-H Stretch	Aldehyde
2459	C ≡ C Stretch	Alkyne
2274	C ≡ N Stretch	Nitrile
1741	C = O Stretch	Carbonyl Group
1541	N-H Bend	Amide
1440	C-H Bend	Alkane
1197	C-O Stretch	Ether or Ester
939	C-H Bend	Alkene
779	C-H Bend	Aromatic
603	C-X Stretch	Halogen Compound (Chlorine, Bromine, etc.)

### 3.2 Gas chromatography-mass spectrometry (GC–MS) analysis

**Fig 3:**
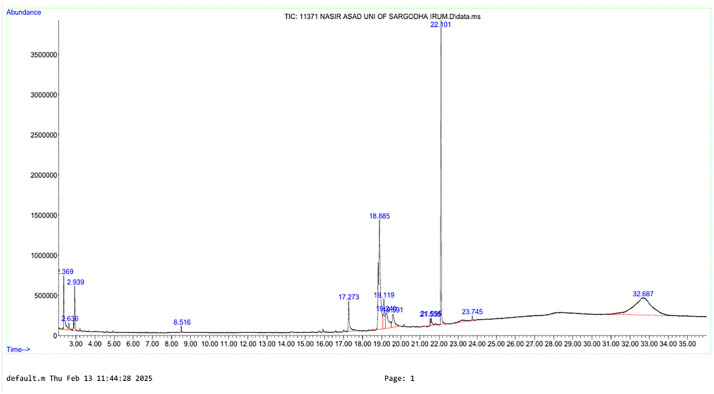
Gas chromatography–mass spectrometry (GC–MS) chromatogram of the methanolic extract of *P. arhizus.*

GC-MS analysis showed that the fungal extract contained more than 12 compounds (Fig 3), and Table 3 provides details of the tentatively identified compounds.

**Table 3:** Bioactive compounds detected by GCMS analysis.

Peak Number	Compound names	Area Percentage (%)	Retention Time (min)
1	N,N-Dimethylacetamide	3.81	2.369
2	m-xylene, o-xylene, p-xylene	0.78	2.636
3	m-xylene, o-xylene, p-xylene	3.61	2.939
4	2-Ethylhexyl acrylate	0.34	8.516
5	n-Hexadecanoic acid (Palmitic acid)	3.58	17.273
6	Oleic acid (9-octadecenoic acid)	21.79	18.885
7	Linoleic acid (9,12-octadecadienoic acid)	4.30	19.119
8	Linoleic acid (9,12-octadecadienoic acid)	5.20	19.240
9	9,12-Octadecadien-1-ol, (Z,Z)	3.45	19.591
10	Linoleic acid (9,12-octadecadienoic acid)	0.26	21.538
11	Linoleic acid (9,12-octadecadienoic acid)	0.82	21.595
12	Bis(2-ethylhexyl) phthalate	16.47	22.101
13	Bis(2-ethylhexyl) terephthalate	1.80	23.745
14	3-(6-Methyl-3-pyridyl)-1,5-diphenyl-2-pyrazoline (pyrazoline) (tentatively identified)	33.80	32.687

The retention times and relative amounts of the main bioactive compounds identified are shown in the chromatogram. These peaks are associated with different secondary metabolites, which can have different biological activities, such as fatty acids, esters, and aromatic compounds. The highest peaks were observed at retention times of 18.885, 22.101, and 32.687 min, indicating the presence of dominant compounds in the extract (Table 3).

### 3.3 Antibacterial activity

The putatively identified pyrazoline derivative (tentatively identified) and octadecanoic acid, along with the *P. arhizus* crude extract, were evaluated against *S. aureus* (ATCC 23235) using the well diffusion method (Table 4). Tetracycline (20 µg/mL) served as a positive control, yielding an inhibition zone of 22.0 ± 0.40 mm. The *P. arhizus* methanolic extract exhibited significant antibacterial activity (20.0 ± 0.30 mm), while pyrazoline and octadecanoic acid produced inhibition zones of 19.0 ± 0.40 mm each. No inhibitory effect was observed in the DMSO negative control as shown in S2 Fig. All assays were conducted in three independent biological replicates. Statistical analysis via one-way ANOVA followed by Tukey’s HSD post-hoc test confirmed significant variance between treatments (F = 48.0, df = 3, p < 0.0001), with the extract showing significantly higher activity than the individual metabolites (p < 0.05). Notably, while these *in vitro* results are consistent with the computational interaction profiles, they do not directly validate efflux pump inhibition as the sole mechanism of action; further mechanistic studies, such as ethidium bromide accumulation assays, are required to confirm this mode of action.

**Table 4:** Antibacterial activity and MIC of *P. arhizus* extract and selected metabolites against *S. aureus* (ATCC 23235).

Sample	Concentration (µg/mL)	Mean Zone of Inhibition (mm) ± SD	MIC (µg/mL)
Tetracycline (Positive Control)	20	22.0 ± 0.40^a^	10
*P. arhizus* Extract	20	20.0 ± 0.30^b^	30
Octadecanoic Acid	20	19.0 ± 0.40^c^	30
Pyrazoline	20	19.0 ± 0.40^c^	40
DMSO (Negative Control)	1% v/v	0.0 ± 0.00	–

*Means followed by different letters (a,b,c) are significantly different according to Tukey’s HSD test (p < 0.05).

The MIC results were consistent with those of the well-diffusion assay, where tetracycline exhibited the highest potency (MIC = 10 µg/mL). The *P. arhizus* extract and octadecanoic acid showed moderate activity (MIC = 30 µg/mL), while pyrazoline was less potent (MIC = 40 µg/mL). These results suggest that the crude extract may exert stronger effects than the isolated metabolites due to potential additive or synergistic interactions. These findings highlight the potential of *P. arhizus* metabolites as antibacterial agents, warranting further mechanistic research against multidrug-resistant *S. aureus* strains. Notably, the antibacterial results align with computational predictions, which is consistent with the favorable binding affinities and stable interaction profiles predicted computationally. However, while these metabolites inhibit bacterial growth, further specific assays are needed to confirm whether this is due to efflux pump inhibition. Although the antibacterial activity observed is consistent with the computational predictions, the mechanism responsible for bacterial growth inhibition remains to be experimentally determined. These findings are consistent with those of prior investigations describing the antibacterial activity of extracts and their metabolites [48–50]^.^

These results are consistent with previous studies on basidiomycota fungi and plant-derived compounds, which have been shown to influence gram-positive and gram-negative bacteria by disrupting their cell walls and producing reactive oxygen species [51] (Table 4). Recently, another study reported similar results [52]. Shrestha et al. (2005) reported that Pisolithus spp. have a broad-spectrum activity against gram-negative and gram-positive bacteria [53]. Similarly, Ameri et al. (2011) showed that the methanolic extracts of *P. albus* have high levels of inhibitory activity against various strains of *S. aureus*, including clinical isolates of methicillin-resistant *S. aureus* (MRSA) [54]. These findings highlight the potential of *P. arhizus* metabolites as natural antibacterial agents and potential efflux pump inhibitor candidates. Further mechanistic research is required on their application against multidrug resistant strains, such as those of *S. aureus*, including detailed efflux pump inhibition assays and synergistic research with established antibiotics.

### 3.4 *In silico* ADMET and drug-likeness profiling

The physicochemical properties and drug-likeness of the identified *P. arhizus* metabolites were evaluated based on Lipinski’s rule of five (S2 Table). Our analysis revealed that most compounds complied with these criteria, with approximately six metabolites showing only one minor Lipinski violation, typically related to cLogP or the number of rotatable bonds, while the remaining candidates exhibited zero violations, indicating favorable drug-like potential [55,56]. Specifically, violations of the cLogP and rotatable bond categories were observed for long-chain lipophilic compounds, which are important considerations for future drug development [57,58].

The metabolites were prioritized based on their balanced physicochemical profiles. For instance, xylene derivatives and long-chain fatty acids exhibit high lipophilicity (cLogP > 6.0), which, while potentially favoring membrane permeation, suggests that their binding interactions might be driven primarily by nonspecific hydrophobic contacts rather than highly directional polar interactions. Conversely, bis(2-ethylhexyl) terephthalate displayed high flexibility (16 rotatable bonds), which may limit its target specificity. In contrast, the pyrazoline derivative displayed an optimal balance of properties: zero Lipinski violations, moderate polarity (TPSA 23.89 Å²), and a balanced cLogP (4.519). This profile, which supports both hydrophobic and polar contributions to target engagement, confirms the pyrazoline derivative as the most robust candidate for downstream, structure-based investigations.

### 3.5 Pharmacokinetic analysis

SwissADME and pkCSM were used to assess the ADMET properties of the *P. arhizus* metabolites (S3 Table). High GI absorption was observed for most metabolites, including the pyrazoline derivative, (tentatively identified) suggesting a good potential for oral administration. In terms of metabolic interactions, the analysis showed that six compounds are inhibitors of CYP1A2, the major cytochrome isoform affected in this investigation. These results were used as developability markers to prioritize candidates for further development of leads, and CYP1A2 inhibition is one of the key pharmacokinetic factors that is an important consideration when considering potential drug-drug interactions [59].

In comparison to the pyrazoline derivative, the fatty acids (e.g., octadecanoic acid and n-hexadecanoic acid) demonstrated high GI absorption but a lower likelihood of metabolic interference, owing to their narrower CYP inhibition profile. In contrast, xylene derivatives were predicted to have low GI absorption, and bis(2-ethylhexyl) terephthalate was predicted to be a substrate for P-glycoprotein (P-gp), suggesting potential efflux in mammalian systems. These collective ADMET predictions helped prioritize the lead candidates, with the pyrazoline derivative being the most balanced lead candidate for downstream structural analysis.

### 3.6 Molecular docking analysis

Molecular docking was performed to determine the binding propensity of the identified metabolites in the methanolic extract to the four major efflux transporters of *S. aureus*, NorA, NorB, NorC, and MepA. All identified metabolites were filtered, and the five compounds that best matched Lipinski’s rule of five were ranked highly and subjected to advanced interaction analysis (S4 and S5 Tables). Compound-dependent affinity differences were indicated by docking scores, with higher (more negative) docking scores indicating stronger binding [60].

**Fig 4:**
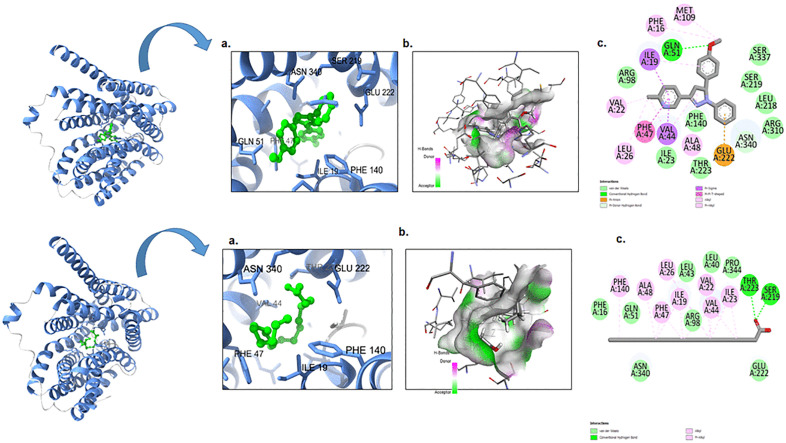
(a) Docked molecular interactions of the top ligand, pyrazoline, and octadecanoic acid of *P. arhizus* with NorA residues.

**Fig 5:**
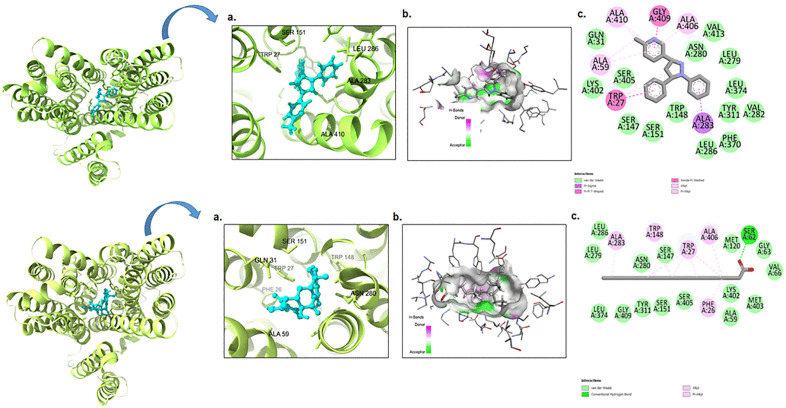
(a) Docked molecular interactions and (b) two-dimensional molecular interactions of the top ligand; pyrazoline and octadecanoic acid of *P. arhizus* with NorB.

**Fig 6:**
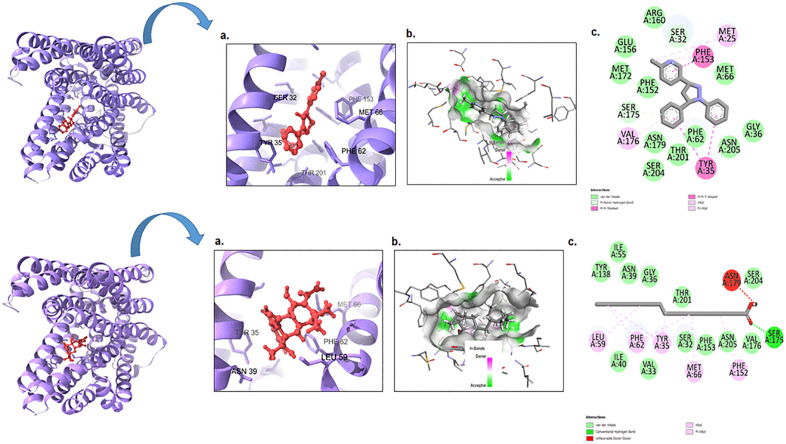
(a) Docked, (b) hydrogen bonding figure, (c) two-dimensional molecular interactions of the top ligand pyrazoline and octadecanoic acid of *P. arhizus* with mepA.

The docking score of pyrazoline was the most promising (−9.3 kcal/mol), indicating the highest potential interaction with NorA. Furthermore, this compound always had the highest binding affinities to the rest of the efflux transporters, with scores of −8.9 kcal/mol for NorB, – 8.4 kcal/mol for NorC, and −8.7 kcal/mol for MepA. A close examination showed that the high affinity of pyrazoline is a phenomenon caused by a wide range of strong intermolecular forces. In NorA, the ligand established a traditional hydrogen bond with GLN51, a Pi-anion and Pi-donor with GLU222 and ASN340, and important Pi-sigma and Pi-Pi T-shaped with ILE19, VAL44, and PHE47 (Fig 4). These complex binding networks were also found with other targets; for example, pyrazoline binding with TRP27 in the π-π stacking in NorB (Fig 5) and TYR35 and PHE153 in MepA (Fig 6) and via π-donor hydrogen bonding with TYR307 and π-π stacking with TRP144 in NorC. Such a broad-spectrum binding profile suggests that tentatively identified pyrazoline derivative may interact favorably with multiple efflux pump targets.

**Fig 7:**
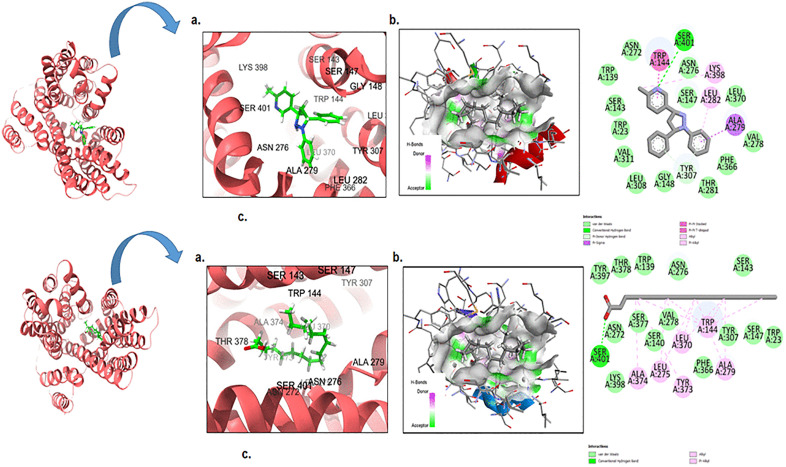
(a) Docked, (b) hydrogen bonding figure, (c) two-dimensional molecular interactions of the top ligand pyrazoline and octadecanoic acid of *P. arhizus* with NorC.

In contrast, fatty acid-type metabolites, such as n-hexadecanoic acid oleic acid and octadecanoic acid, had moderate scores in docking against NorA (−5.8 to −6.5 kcal/mol) and a weak moderate affinity with NorB, NorC, and MepA (−5.0 to −5.6 kcal/mol). Interaction profiling of the secondary lead, octadecanoic acid, showed that the interactions mainly occur between the target proteins and the secondary lead via nonspecific hydrophobic and alkyl/Pi-alkyl contacts. For example, octadecanoic acid in NorA interacts with ILE19, VAL22, ILE23, LEU26, VAL44, PHE47, ALA48, and PHE140, with only two additional standard hydrogen bonds (Figs 4–6). It was negatively in conflict with ASN179 as a donor of MepA. In NorC, octadecanoic acid interacts primarily through van der Waals forces with residues such as TRP23, TRP139, and ASN272, supplemented by conventional hydrogen bonding with SER401 (Fig 7)**.**

Together, these findings indicate that, when compared with the pyrazoline lead, long-chain lipophilic metabolites exhibit less favorable predicted binding and are mostly dependent on nonspecific hydrophobic interactions, as opposed to the highly directional, Pi-driven binding found within the transporter cavities of the pyrazoline complexes.

### 3.7 Molecular dynamics simulation: Ligand stability analysis

**Fig 8:**
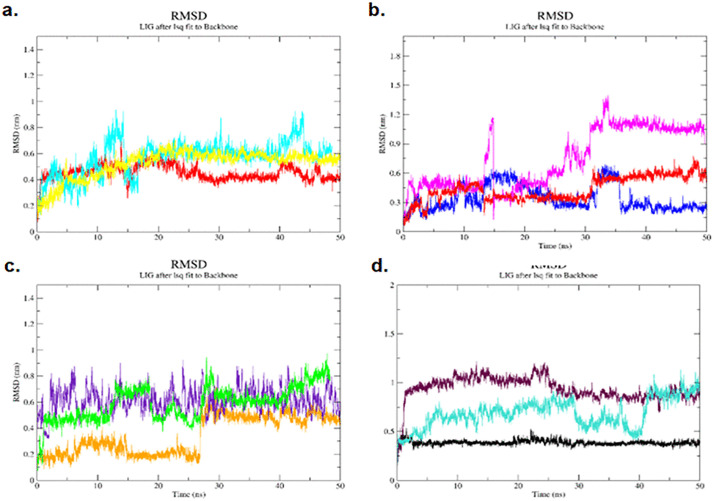
Ligand-specific Root Mean Square Deviation (RMSD) profiles. The plots illustrate the structural stability and conformational convergence of pyrazoline derivative, octadecanoic acid and Tetracycline (control) within the binding pockets of **(a)** NorA, **(b)** NorB, and **(c)** MepA, **(d)** NorC efflux pumps over a 50 ns simulation.

Root mean square deviation (RMSD) analysis was used to quantify the structural stability of the docked complexes during the 50 ns simulation period, with all least squares of the trajectories fitted to their respective protein backbones (Fig 8a–8d). This analysis provides a measure of the conformational fluctuations and binding stability within the efflux pump channels.

For the NorA system (Fig 8a), the pyrazoline derivative (red) exhibited stable deviations, stabilizing at approximately 0.4 nm, whereas the octadecanoic acid (cyan) and tetracycline control (yellow) showed higher RMSD fluctuations, indicating greater mobility at the binding site. In the NorB system (Fig 8b), the pyrazoline derivative (blue) remained stable, with RMSD values of approximately 0.3 nm after reaching equilibrium. Although the conformation of octadecanoic acid (magenta) fluctuated markedly after 30 ns, the pyrazoline moiety remained firmly bound, indicating favorable structural stability during the simulation. Regarding the MepA system (Fig 8c), the pyrazoline derivative (orange) showed minimal deviations and stabilized at approximately 0.2 nm, whereas the octadecanoic acid (indigo) and tetracycline control (green) exhibited a wider range of fluctuations (0.4–0.8 nm), suggesting a less restricted binding mode. Finally, for the NorC system (Fig 8d), the pyrazoline derivative (black) displayed the most stable trajectory with minimal fluctuations (approximately 0.4 nm), whereas the octadecanoic acid (cyan) and tetracycline control (purplish) showed higher RMSD profiles, particularly in the latter half of the simulation.

These MD results show that the pyrazoline derivative rapidly reached equilibrium and maintained stable interactions across all four efflux transporters. The minimal RMSD values observed for the pyrazoline complexes across all targets suggest a precise and rigid binding mode, which may favor stable protein–ligand interactions. The higher fluctuations observed for octadecanoic acid and tetracycline suggest a more dynamic interaction, potentially allowing for greater ligand displacement within the transporter pocket than that in the stabilized pyrazoline complex [61].

#### Root Mean Square Fluctuation (RMSF) profiles.

**Fig 9:**
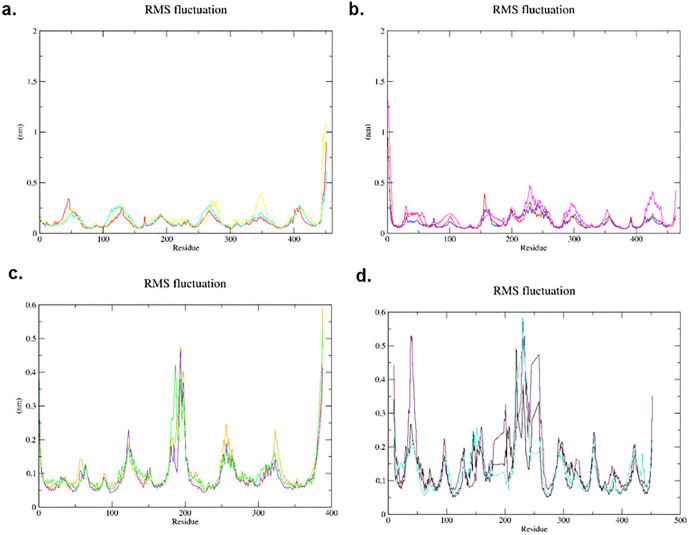
Per-residue RMSF (Root Mean Square Fluctuation) profiles. These are plotted for the local protein backbone flexibility of all four efflux pumps. **(a)** MepA complexes: pyrazoline derivative (red), octadecanoic acid (cyan), and tetracycline (yellow). **(b)** NorB complexes: pyrazoline derivative (blue), octadecanoic acid (magenta), and tetracycline (red). **(c)** Recovery of NorA complexes: pyrazoline derivative (orange), octadecanoic acid (indigo), and tetracycline (green). and **(d)** NorC complexes: pyrazoline derivative (black), octadecanoic acid (purplish), and Tetracycline (turquoise).

The residue-level fluctuations were analyzed to assess the local backbone flexibility across the four systems (Fig 9a–9d), which consistently demonstrated that the pyrazoline derivative and octadecanoic acid provided superior stabilization compared to the tetracycline control.

In the NorA (Fig 9a) and MepA (Fig 9c) systems, the tetracycline reference (yellow/green) showed greater flexibility within its loops, whereas the complexes of pyrazoline (red/orange) and octadecanoic acid (cyan/indigo) had lower RMSF values (typically < 0.4 nm). In the NorB system (Fig 9b), the pyrazoline derivative (blue) showed the greatest ability to reduce fluctuations, with most peaks below 0.3 nm, suggesting a tighter structure of the transmembrane helices than octadecanoic acid (magenta) or the tetracycline reference (red). The pyrazoline-bound complex (black) in the NorC system (Fig 9d) had more restricted mobility over critical functional residues than the greater mobility observed in the octadecanoic acid (cyan) and tetracycline (purple) complexes.

In molecular docking, hydrogen bonds are necessary to maintain the stability of the ligand–protein complex, whereas alkyl interactions increase the overall binding affinity [55]. In addition to residue flexibility, hydrogen bond persistence was monitored throughout the 50 ns simulations (S3(a)–S3(d) Fig). The pyrazoline derivative consistently maintained 2–4 stable hydrogen bonds across all four transporters, whereas tetracycline control and octadecanoic acid displayed greater volatility and lower bond occupancy, particularly in NorB and MepA. Overall, the fungal metabolites exhibited lower residue fluctuations than tetracycline, suggesting enhanced stabilization of transporter dynamics during the simulations [62].

#### Structural Compactness (Rg) and Solvent Accessible Surface Area (SASA).

**Fig 10:**
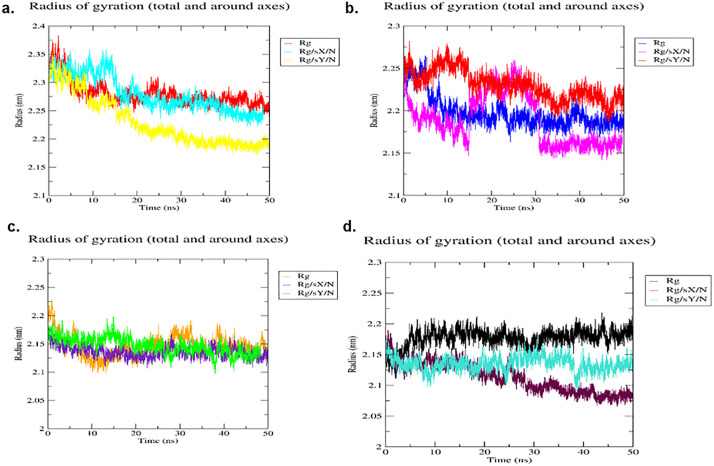
Radius of gyration (Rg) over 50 ns molecular dynamics simulations. Trajectories illustrate the overall structural compactness of **(a)** NorA, **(b)** NorB, **(c)** MepA and **(d)** NorC efflux pumps in complex with the derivative of pyrazoline, octadecanoic acid, and the control antibiotic tetracycline.

Radius of gyration (Rg) and solvent-accessible surface area (SASA) were calculated to verify the overall structural compactness and folding stability of the different protein–ligand complexes along the 50 ns simulation trajectory (Fig 10a–10d). With respect to the general trends in the evolution of the Rg values, convergence and stability indicate that the system reaches a compact and structurally stable state, which is also an important element for obtaining consistent results in further computational examinations [61,63].

In all of the systems, the pyrazoline derivative and octadecanoic acid converged consistently. For the complexes in the NorA system (Fig 10a), the complexes were stabilized around 2.25–2.27 nm, with the pyrazoline derivative (orange) being more stable than the tetracycline control (yellow). In the NorB system (Fig 10b), the pyrazoline derivative (blue) was seen to reach a stable conformation of ~2.18 nm, whereas the tetracycline control (red) exhibited higher, more dispersed values (around 2.22 nm), indicating a more open conformation. In the MepA system (Fig 10c) both fungal metabolites clustered around ~2.13 nm with the control (yellow) showing more variation. The profile of the NorC system (Fig 10d) remained stable at ~2.18 nm, whereas, the pyrazoline complex (black) exhibited a more stable profile at ~2.18 nm as compared to the octadecanoic acid (cyan, ~ 2.13 nm) and the control (purplish, ~ 2.08 nm).

#### Solvent Accessible Surface Area (SASA).

**Fig 11:**
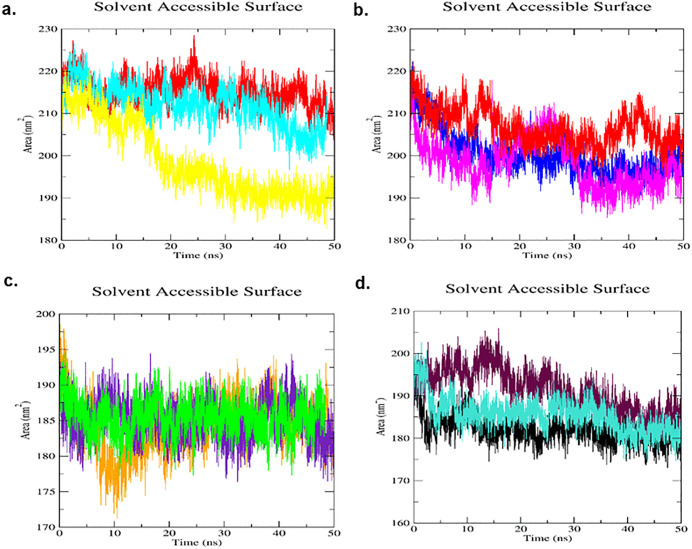
Solvent Accessible Surface Area (SASA) over 50 ns molecular dynamics simulations. The trajectories illustrate the solvent exposure of the **(a)** NorA, **(b)** NorB, **(c)** MepA and **(d)** NorC efflux pumps when complexed with the pyrazoline derivative, octadecanoic acid, and the control antibiotic Tetracycline.

The well-maintained SASA profile over time suggests that the complex is stable. Similarly, studies have shown that a better SAS indicates that the binding area of the drug can move and adjust while maintaining stable contact with the target [64]. The pyrazolyl derivative (orange) and octadecanoic acid (indigo) in the NorA complexes (Fig 11a) showed a stable SASA profile, with the modes oscillating within the 175–190 nm^2^ range. The tetracycline control (green) had similar values but with a larger variance, indicating a more dynamic protein–solvent interface. In the NorB system, the pyrazoline lead (blue; Fig 11b) had a well-equilibrated SASA (~ 195–200 nm^2^), which exhibited superior structural stability compared to the tetracycline control (red), with greater surface exposure (up to 210–220 nm^2^). Octadecanoic acid (magenta) also exhibited a compact profile that spanned nearly 190 nm^2^. The MepA system, pyrazoline (red), and octadecanoic acid (cyan; Fig 11c) exhibited stable SASA trajectories (205–215 nm^2^), whereas the tetracycline control (yellow) in the system indicated high structural changes, and the level decreased rapidly (185–190 nm^2^) in 20 ns. Furthermore, in the NorC system (Fig 11d), the pyrazoline derivative (black) achieved the most stable equilibrium at ~180 nm², contrasting with the higher solvent exposure observed for both the octadecanoic acid (cyan, ~ 185 nm²) and tetracycline (purplish, ~ 190–195 nm²) complexes.

Thus, RMSD, RMSF, Rg, and SASA analyses confirmed that pyrazoline lead and octadecanoic acid consistently stabilized all four efflux pumps. In contrast to the dynamic and structurally dispersed behavior of the tetracycline control, both fungal metabolites were associated with more stable conformational states during simulation. These findings suggest that both ligands stabilize the efflux pump structures during simulation; however, experimental studies are required to determine whether these effects translate into functional inhibition. With this structural stability in place, we used the MM-GBSA method to determine the thermodynamic binding affinity and energetic favorability of these complexes.

### 3.8 Binding free energy estimation using prime MM-GBSA

**Fig 12:**
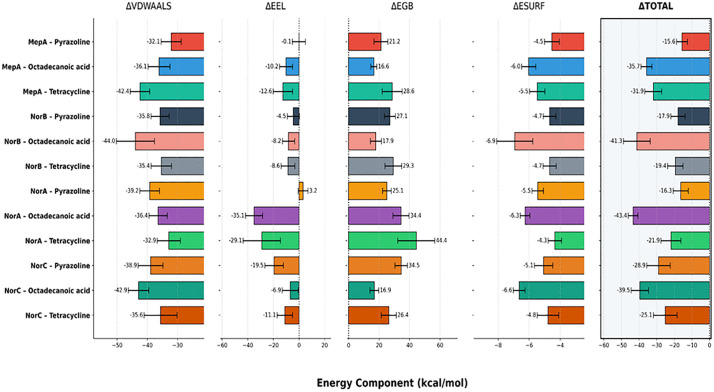
MM/GBSA binding free energy contributions of selected ligands complexed with the NorA, NorB, MepA, and NorC efflux pump proteins of *S. aureus.* The graph displays the decomposed energy components (van der Waals, electrostatic, polar, and nonpolar solvation energies) for three ligands: pyrazoline, octadecanoic acid, and tetracycline (used as reference).

The MM-GBSA binding free energy (ΔG_bind)_ values of the selected ligands were computed to evaluate their relative binding strengths, and the results are summarized in Fig 12 and S6 Table. Octadecanoic acid exhibited the most favorable binding free energies across all transporters, with values ranging from −35.73 to −43.37 kcal/mol. The tetracycline control exhibited a strong binding profile (ranging between −21.86 and −31.89 kcal/mol), and the pyrazoline derivative exhibited moderate and consistent binding affinities (NorA −16.33 kcal/mol; NorB −17.87 kcal/mol; MepA −15.58 kcal/mol; and NorC −28.94 ± 6.68 kcal/mol) (Fig 11). Notably, the pyrazoline lead had a consistent thermodynamic signature in all four transporters, implying that it was strongly bound in a stable binding mode of hydrophobic stabilization. Although the overall binding energy of octadecanoic acid was the highest, the performance of the tentatively identified pyrazoline derivative is indicative of its potential as a multitarget efflux pump inhibitor candidate because of its potential to engage many different efflux pumps.

### 3.9 Component analysis (PCA) and Dynamic cross-correlation matrix (DCCM) analysis

The conformational landscape and internal dynamic coupling of NorA, NorB, MepA, and NorC efflux pumps in the presence of the lead ligand pyrazoline and the secondary metabolite octadecanoic acid were measured to characterize the global motions and patterns of residue-residue coupling.

**Fig 13:**
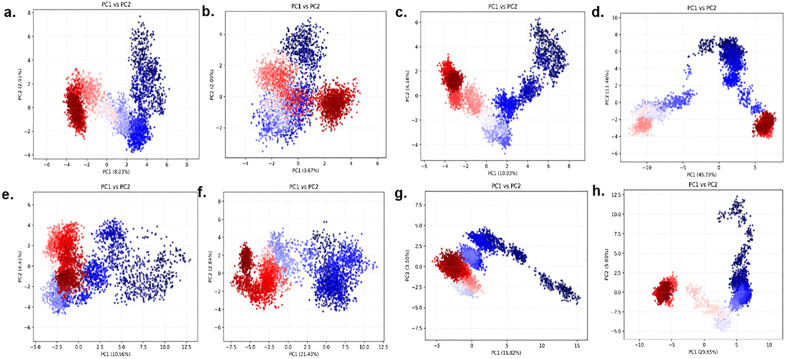
Principal Component Analysis (PCA) of protein ligand complexes. Panels display **(a)** NorA–pyrazoline; **(b)** NorA–Octadecanoic acid; **(c)** NorB–pyrazoline; **(d)** NorB–Octadecanoic acid; **(e)** MepA–pyrazoline; and **(f)** MepA–Octadecanoic acid, **(g)** NorC–pyrazoline; and **(h)** NorC–octadecanoic acid.

Principal component analysis (PCA) was used to project the conformational subspace of the protein-ligand complexes. The simulation trajectories were projected onto the two principal components (PC1 vs. PC2) to identify clear conformational clustering, indicating that ligand binding restricts the protein to a limited number of functional states (Fig 13a–13h). The associated scree plots showed that the eigenvalues decayed rapidly, validating that a few principal motions explained most of the structural variance (S4 Fig). The percentage variance explained by PC1 differed between systems: 8.03% (MepA-pyrazoline), 2.09% (MepA-octadecanoic acid), 10.03% (NorB-pyrazoline), 11.46% (NorB-octadecanoic acid), 10.56% (NorA-pyrazoline), 2.84% (NorA-octadecanoic acid), 11.82% (NorC-pyrazoline), and 29.65% (NorC-octadecanoic acid). The sharp decrease in these plots indicates that the major collective motions were highly localized. Moreover, the pyrazoline complexes were consistently stabilized in the distribution of trajectories, unlike the more extensive conformational search observed in the fatty acid-bound systems.

#### Dynamic Cross-Correlation Matrix (DCCM) analysis.

**Fig 14:**
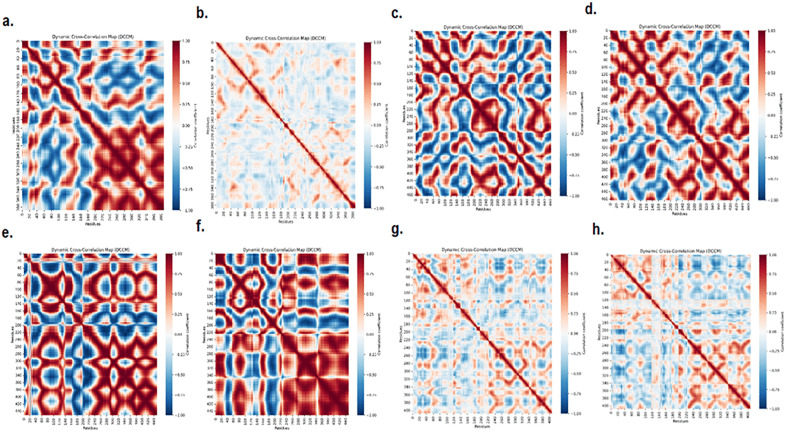
Dynamic cross-correlation matrix (DCCM) analysis of protein-ligand complexes. Panels display: **(a)** NorA–pyrazoline; **(b)** NorA–Octadecanoic acid; **(c)** NorB -pyrazoline; **(d)** NorB–Octadecanoic acid; **(e)** MepA–pyrazoline; and **(f)** MepA–Octadecanoic acid, **(g)** NorC–pyrazoline; and **(h)** NorC–octadecanoic acid.

The DCCM analysis also provided more information on the effect of these ligands on the long-range residue-residue coupling (Fig 14a–14h). Enhanced regions of both coupled (red) and anticoupled (blue) movements were also observed in the pyrazoline-bound complexes (Fig 14a, 14c, 14e and 14g), with well-defined localized correlation clusters. This indicates that tentatively identified pyrazoline derivative binding stabilizes a highly coordinated conformational state, probably by limiting the mobility of the transmembrane helices that participate in the rocking motion transport process of the protein. In contrast, the complexes with octadecanoic acid (Fig 14b, 14d, 14f and 14h) exhibited a more spread-out and dampened correlation pattern, with a predominant predominance of uncorrelated and neutral motions. This shows that although octadecanoic acid interacts with the pumps, it has less influence on the concerted and long-range movements required for functional transport.

These findings collectively imply that the pyrazoline derivative may act as a more effective modulator of interdomain communication, reinforcing its potential as a potential modulator of structural transitions associated with efflux pump function. Although the observed antibacterial activity and *in silico* results are promising, specific assays, such as ethidium bromide (EtBr) accumulation or antibiotic potentiation assays, are required to definitively confirm efflux pump inhibition and rule out other mechanisms of action.

## 4. Discussion

In this study, metabolites from *P. arhizus* were evaluated using an integrated experimental–computational workflow against *S. aureus* efflux pumps. The well-diffusion assay demonstrated significant antibacterial activity, consistent with the reported broad-spectrum antimicrobial properties of this fungal genus [52,65]. The observed antibacterial efficacy of *P. arhizus* extracts against *S. aureus* suggests that the constituent metabolites may represent promising scaffolds for the development of inhibitors targeting efflux pumps, such as NorA, NorB, NorC, and MepA, which are key contributors to multidrug resistance [20,66]**.** These membrane-bound transporters act as critical defense mechanisms by expelling antibiotics and other toxic compounds from bacterial cells, thereby reducing their intracellular concentrations and efficacy**.** Recent studies have further suggested that efflux pumps, such as NorA, can synergistically enhance virulence and biofilm formation, highlighting their multifaceted role in *S. aureus* pathogenesis [67]. By computationally evaluating these fungal compounds against multiple efflux pumps (NorA, NorB, NorC, and MepA), this study highlights their potential as multitarget efflux pump inhibitor candidates. This study bridges the gap between traditional knowledge of fungal bioactivity and modern drug discovery, highlighting *P. arhizus* as a promising source of bioactive metabolites for future antimicrobial research [68].

The interactions of these substances with *S. aureus* proteins (NorA, NorB, NorC, and MepA) were examined using molecular docking analysis. These metabolites exhibited favorable predicted interactions with efflux pump proteins and warrant further experimental investigation. The extensive interaction network formed by the tentatively identified pyrazoline may contribute to its favorable predicted binding affinity [69,70]. The combination of GC-MS analysis, ADMET profiling, molecular docking, and molecular dynamics simulation in this study showed that a number of fungal-derived metabolites, especially 3-(6-methyl-3-pyridyl)-1,5-diphenyl-2-pyrazoline (tentatively identified), have favorable pharmacokinetic properties, strong predicted binding affinities, and stable protein–ligand interactions. Because this identification relies on GC–MS library matching, further analytical confirmation, such as NMR or high-resolution mass spectrometry, is required for definitive structural elucidation. These results indicate that *P. arhizus* metabolites may be useful scaffolds for establishing potential efflux pump inhibitor candidates against multidrug-resistant *S. aureus*. Although the computational and antibacterial findings are encouraging, further *in vitro* and *in vivo* studies are required to validate efficacy, mechanism of action, and safety. This study highlights the potential of fungal secondary metabolites as novel pharmacophores and the application of structure-based drug discovery methods for antimicrobial resistance.

## 5. Conclusions

In this study, an *in silico* framework was applied to identify and characterize bioactive compounds from *P. arhizus* with predicted interactions toward the efflux pump proteins of *S. aureus*, a major pathogen of bovine mastitis. The integration of GC-MS analysis, ADMET profiling, molecular docking, and molecular dynamics simulations revealed that several fungal-derived metabolites, particularly the tentatively identified pyrazoline derivative, were predicted to exhibit favorable pharmacokinetic properties, strong binding affinities, and stable protein–ligand interactions. These findings suggest that selected compounds of *P. arhizus* may serve as promising scaffolds for developing potential efflux pump inhibitor candidates to combat multidrug resistance in *S. aureus*. Although these computational results provide preliminary evidence supporting further investigation of their therapeutic potential, further *in vitro* and *in vivo* validation is warranted to confirm antibacterial activity and safety. This study underscores the value of fungal secondary metabolites as a reservoir of novel pharmacophores and highlights the relevance of structure-based drug discovery strategies in addressing antimicrobial resistance**.**

## Supporting information

S1 Fig Crystallographic structure of the x-ray crystallographic structure of the *Staphylococcus aureus* Quinolone resistance proteins (a) NorA (AF ID: AF-Q53459-F1-model_v4) (b) NorB, (c) NorC, (d) MepA respectively downloaded from the Alphafold database. (DOCX)

S2 Fig Anti-bacterial activity *against Staphylococcus aureus* (ATCC 23235), (A) Tetracycline (Positive control), (B) *P. arhizus* extract, (C) Octadecanoic acid, (D) Pyrazoline and (E) DMSO (Negative control). (DOCX)

S3 Fig Number of intermolecular hydrogen bonds over 50 ns molecular dynamics simulations. The plots display the dynamic hydrogen bonding interactions between the binding pockets of four distinct efflux pump proteins and their respective ligands: 3-(6-Methyl-3-pyridyl)-1,5-diphenyl-2-pyrazoline, octadecanoic acid, and the control antibiotic Tetracycline. (a) MepA complexes: pyrazoline derivative (red), octadecanoic acid (cyan), and Tetracycline (yellow). (b) NorB complexes: pyrazoline derivative (blue), octadecanoic acid (magenta), and Tetracycline (red). (c) NorA complexes: pyrazoline derivative (orange), octadecanoic acid (indigo), and Tetracycline (green). (d) NorC complexes: pyrazoline derivative (black), octadecanoic acid (maroon), and Tetracycline (turquoise).
(DOCX)

S4 Fig The lower panels show the corresponding Scree plots depicting the percentage of total variance captured by the top-ranked eigenvalues. Complexes: (a) NorA–pyrazoline; (b) NorA–Octadecanoic acid; (c) NorB -pyrazoline; (d) NorB–Octadecanoic acid; (e) Mep- pyrazoline; and (f) MepA–Octadecanoic acid, (g) NorC- pyrazoline; and (h) NorC- Octadecanoic acid.
(DOCX)

S1 Table Compounds derived from *Pisolithus arhizus.* (DOCX)

S2 Table PASS analysis results of the 12 *Pisolithus arhizus* compounds (Lipinski rule of five). (DOCX)

S3 Table Pharmacokinetic features of selected mycoligands from *Pisolithus arhizus.* (DOCX)

S4 Table Molecular docking results of selected ligands of *P. arhizus’s*, their binding affinity values and their bonding interactions with residues. (DOCX)

S5 Table Molecular docking results of selected ligands of *P. arhizus*, their binding affinity values, and bonding interactions with residues. (DOCX)

S6 TableSummary of MM-GBSA binding free energy components ($\Delta G_{bind}$) calculated for the selected efflux pump–ligand complexes.(DOCX)
