# Supplementary material for: Computational and experimental evaluation of Pisolithus arhizus metabolites targeting major efflux pumps of mastitis-associated Staphylococcus aureus
Source: PLoS One. 2026 Jul 16;21(7):e0354013. doi: 10.1371/journal.pone.0354013 (PMC13374981; doi:10.1371/journal.pone.0354013)
Supplement: S1 Fig — (DOCX) [file pone.0354013.s001.docx]

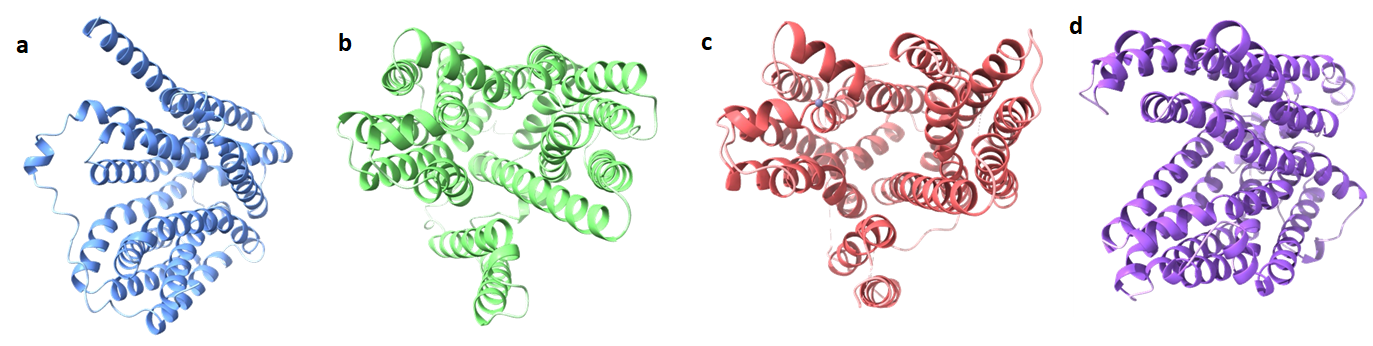


**Figure S1.** Crystallographic structure of the x-ray crystallographic structure of the *Staphylococcus aureus* Quinolone resistance proteins (a) NorA (AF ID: AF-Q53459-F1-model_v4) (b) NorB , (c) NorC, (d) MepA respectively downloaded from the Alphafold database.
