# Supplementary material for: Computational and experimental evaluation of Pisolithus arhizus metabolites targeting major efflux pumps of mastitis-associated Staphylococcus aureus
Source: PLoS One. 2026 Jul 16;21(7):e0354013. doi: 10.1371/journal.pone.0354013 (PMC13374981; doi:10.1371/journal.pone.0354013)
Supplement: S2 Fig — (DOCX) [file pone.0354013.s002.docx]

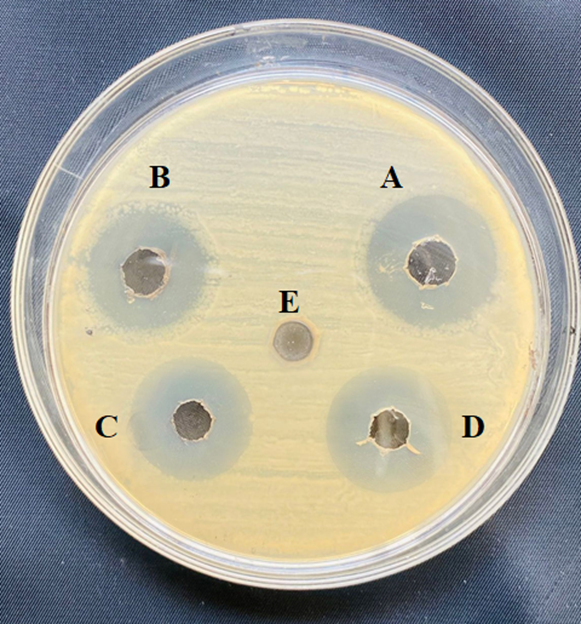


**Figure S2.** Anti-bacterial activity *against Staphylococcus aureus* (ATCC 23235), (A) Tetracycline (Positive control), (B) *P. arhizus* extract, (C) Octadecanoic acid, (D) Pyrazoline and (E) DMSO (Negative control)
