# Supplementary material for: Computational and experimental evaluation of Pisolithus arhizus metabolites targeting major efflux pumps of mastitis-associated Staphylococcus aureus
Source: PLoS One. 2026 Jul 16;21(7):e0354013. doi: 10.1371/journal.pone.0354013 (PMC13374981; doi:10.1371/journal.pone.0354013)
Supplement: S3 Fig — The plots display the dynamic hydrogen bonding interactions between the binding pockets of four distinct efflux pump proteins and their respective ligands: 3-(6-Methyl-3-pyridyl)-1,5-diphenyl-2-pyrazoline, octadecanoic acid, and the control antibiotic Tetracycline. (a) MepA complexes: pyrazoline derivative (red), octadecanoic acid (cyan), and Tetracycline (yellow). (b) NorB complexes: pyrazoline derivative (blue), octadecanoic acid (magenta), and Tetracycline (red). (c) NorA complexes: pyrazoline derivative (orange), octadecanoic acid (indigo), and Tetracycline (green). (d) NorC complexes: pyrazoline derivative (black), octadecanoic acid (maroon), and Tetracycline (turquoise). (DOCX) [file pone.0354013.s003.docx]

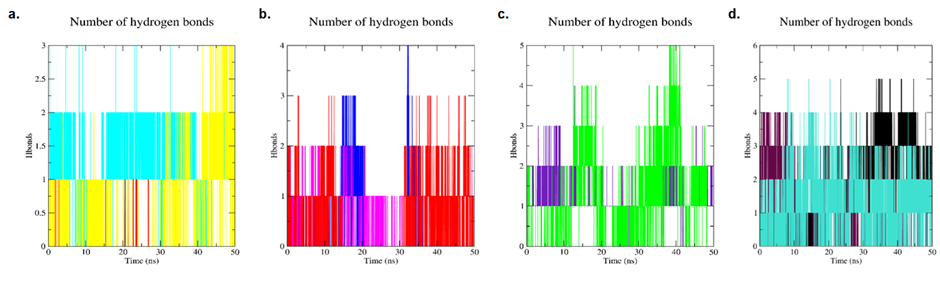


**Figure S3.** Number of intermolecular hydrogen bonds over 50 ns molecular dynamics simulations. The plots display the dynamic hydrogen bonding interactions between the binding pockets of four distinct efflux pump proteins and their respective ligands: 3-(6-Methyl-3-pyridyl)-1,5-diphenyl-2-pyrazoline, octadecanoic acid, and the control antibiotic Tetracycline. (a) MepA complexes: pyrazoline derivative (red), octadecanoic acid (cyan), and Tetracycline (yellow). (b) NorB complexes: pyrazoline derivative (blue), octadecanoic acid (magenta), and Tetracycline (red). (c) NorA complexes: pyrazoline derivative (orange), octadecanoic acid (indigo), and Tetracycline (green). (d) NorC complexes: pyrazoline derivative (black), octadecanoic acid (maroon), and Tetracycline (turquoise).
