# Supplementary material for: Computational and experimental evaluation of Pisolithus arhizus metabolites targeting major efflux pumps of mastitis-associated Staphylococcus aureus
Source: PLoS One. 2026 Jul 16;21(7):e0354013. doi: 10.1371/journal.pone.0354013 (PMC13374981; doi:10.1371/journal.pone.0354013)
Supplement: S4 Fig — Complexes: (a) NorA–pyrazoline; (b) NorA–Octadecanoic acid; (c) NorB -pyrazoline; (d) NorB–Octadecanoic acid; (e) Mep- pyrazoline; and (f) MepA–Octadecanoic acid, (g) NorC- pyrazoline; and (h) NorC- Octadecanoic acid. (DOCX) [file pone.0354013.s004.docx]

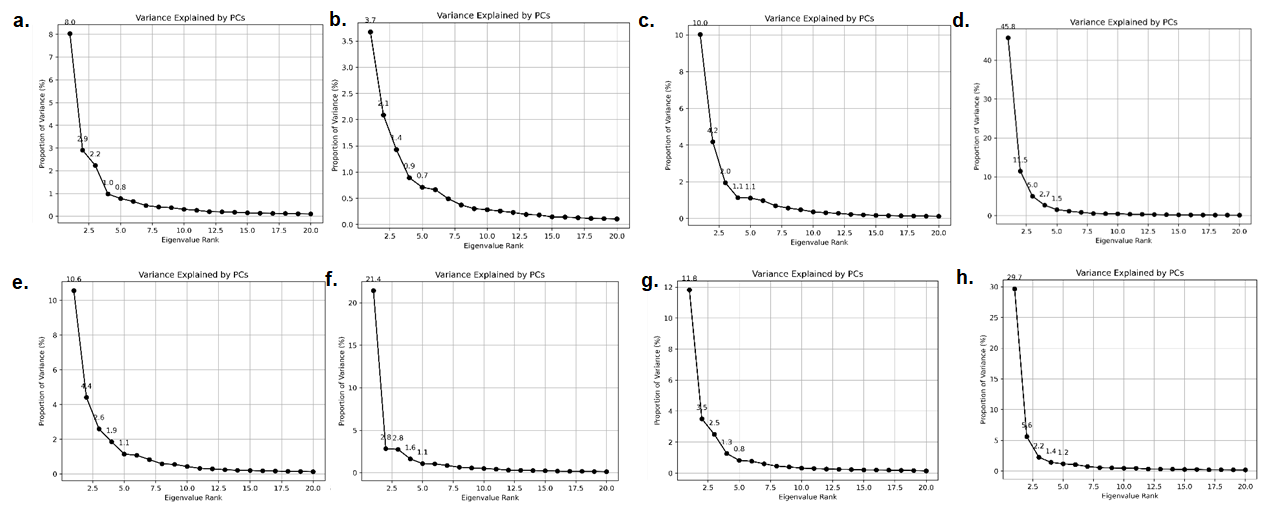


**Figure S4.** The lower panels show the corresponding Scree plots depicting the percentage of total variance captured by the top-ranked eigenvalues. Complexes: (a) NorA–pyrazoline; (b) NorA–Octadecanoic acid; (c) NorB -pyrazoline; (d) NorB–Octadecanoic acid; (e) Mep- pyrazoline; and (f) MepA–Octadecanoic acid, (g) NorC- pyrazoline; and (h) NorC- Octadecanoic acid,
