# Supplementary material for: Computational and experimental evaluation of Pisolithus arhizus metabolites targeting major efflux pumps of mastitis-associated Staphylococcus aureus
Source: PLoS One. 2026 Jul 16;21(7):e0354013. doi: 10.1371/journal.pone.0354013 (PMC13374981; doi:10.1371/journal.pone.0354013)
Supplement: S1 Table — (DOCX) [file pone.0354013.s005.docx]

**Table S1.** Compounds derived from *Pisolithus arhizus.*

| Compound name | PubChem CID | Molecular Formula | Molecular Weigth | Structure |
| --- | --- | --- | --- | --- |
| N,N-Dimethylacetamide | 31374 | C_4_H_9_NO | 78.11g/mol | 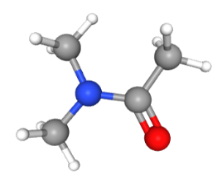 |
| Benzene, 1,3-dimethyl (m-xylene) | 7929 | C_8_H_10_ | 106.16g/mol | 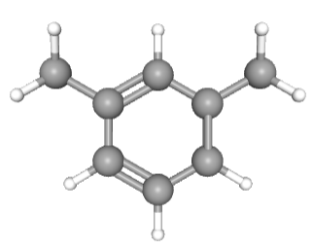 |
| o-Xylene | 7237 | C_8_H_10_ | 106.16g/mol | 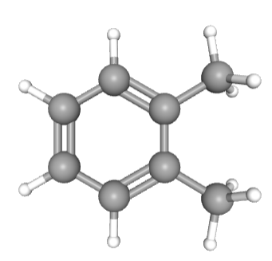 |
| 2-Ethylhexyl acrylate | 7636 | C_11_H_20_O_2_ | 184.27g/mol | 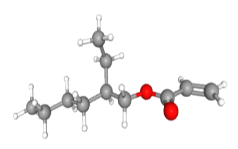 |
| n-Hexadecanoic acid | 985 | C_16_H_32_O_2_ | 256.42g/mol | 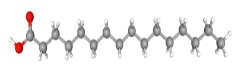 |
| Oleic acid (9-octadecenoic acid) | 637517 | C_18_H_34_O_2_ | 282.5g/mol | 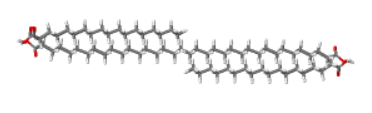 |
| Octadecanoic acid | 5281 | C_18_H_36_O_2_ | 284.5g/mol | 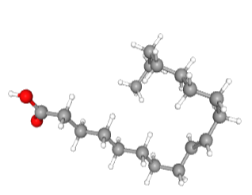 |
| 9,12-Octadecadienoic acid (Z,Z) | 5280450 | C_18_H_32_O_2_ | 280.4g/mol | 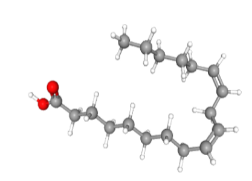 |
| 9,12-Octadecadien-1-ol, (Z,Z) | 5365682 | C_18_H_34_O | 266.5g/mol | 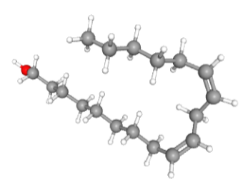 |
| Bis (ethylhexyl)phthalate | 8343 | C_24_H_38_O_4_ | 390.6 g/mol | 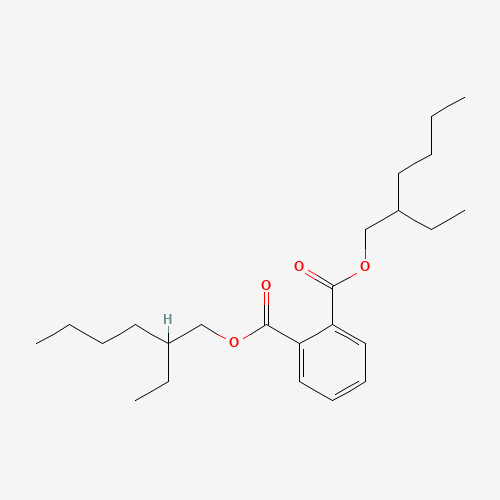 |
| Bis(2-ethylhexyl) terephthalate | 22932 | C_24_H_38_O_4_ | 390.6g/mol | 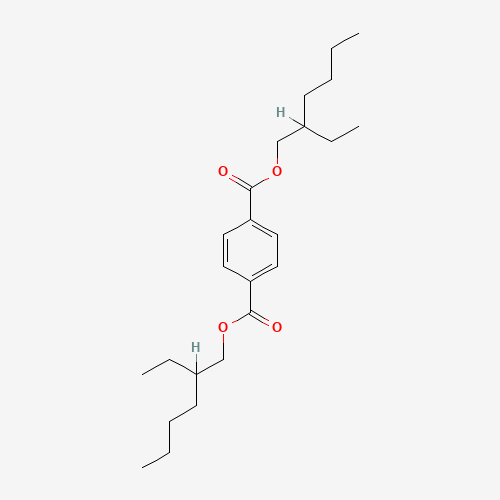 |
| 3-(6-Methyl-3-pyridyl)-1,5-diphenyl-2-pyrazoline | 628595 | C_21_H_19_N_3_ | 313.4g/mol | 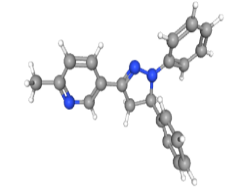 |
