# Supplementary material for: Computational and experimental evaluation of Pisolithus arhizus metabolites targeting major efflux pumps of mastitis-associated Staphylococcus aureus
Source: PLoS One. 2026 Jul 16;21(7):e0354013. doi: 10.1371/journal.pone.0354013 (PMC13374981; doi:10.1371/journal.pone.0354013)
Supplement: S2 Table — (DOCX) [file pone.0354013.s006.docx]

**Table S2.** PASS analysis results of the 12 *Pisolithus arhizus* compounds (Lipinski rule of five).

| Compounds | Rotatable bonds | H-bond acceptors | H-bond donors | TPSA (Å²) | cLogP | Lipinski Violations |
| --- | --- | --- | --- | --- | --- | --- |
| N,N-Dimethylacetamide | 1 | 1 | 0 | 20.31 | -0.0789 | 0 |
| m-xylene | 0 | 0 | 0 | 0.00 | 2.3474 | 0 |
| o-Xylene | 0 | 0 | 0 | 0.00 | 2.3474 | 0 |
| 2-Ethylhexyl acrylate | 8 | 2 | 0 | 26.30 | 3.2943 | 0 |
| n-Hexadecanoic acid | 14 | 2 | 1 | 37.30 | 6.0625 | 1 |
| Oleic acid (9-octadecenoic acid) | 15 | 2 | 1 | 37.30 | 6.7191 | 1 |
| Octadecanoic acid | 16 | 2 | 1 | 37.30 | 6.9713 | 1 |
| 9,12-Octadecadienoic acid (Z,Z) | 14 | 2 | 1 | 37.30 | 6.4669 | 0 |
| 9,12-Octadecadien-1-ol, (Z,Z) | 14 | 1 | 1 | 20.23 | 6.8165 | 0 |
| Bis(2-ethylhexyl)phthalate | 16 | 4 | 0 | 52.60 | 7.2788 | 1 |
| Bis(2-ethylhexyl) terephthalate | 16 | 4 | 0 | 52.60 | 7.2788 | 1 |
| 3-(6-Methyl-3-pyridyl)-1,5-diphenyl-2-pyrazoline | 3 | 2 | 0 | 28.49 | 4.519 | 0 |
