# Supplementary material for: Computational and experimental evaluation of Pisolithus arhizus metabolites targeting major efflux pumps of mastitis-associated Staphylococcus aureus
Source: PLoS One. 2026 Jul 16;21(7):e0354013. doi: 10.1371/journal.pone.0354013 (PMC13374981; doi:10.1371/journal.pone.0354013)
Supplement: S3 Table — (DOCX) [file pone.0354013.s007.docx]

**Table S3.** Pharmacokinetic features of selected mycoligands from *Pisolithus arhizus*.

| Name | GI absorption | BBB permeant | P-gp substrate | CYP1A2 inhibitor | CYP2C19 inhibitor | CYP2C9 inhibitor | CYP2D6 inhibitor | CYP3A4 inhibitor | Log *K*_p_ (skin permeation) |
| --- | --- | --- | --- | --- | --- | --- | --- | --- | --- |
| N,N-Dimethylacetamide | Low | No | No | No | No | No | No | No | -7.38 cm/s |
| m-xylene | Low | Yes | No | No | No | No | No | No | -4.68 cm/s |
| o-Xylene | Low | Yes | No | No | No | No | No | No | -4.73 cm/s |
| 2-Ethylhexyl acrylate | High | Yes | No | No | No | No | No | No | -4.70 cm/s |
| n-Hexadecanoic acid | High | Yes | No | Yes | No | Yes | No | No | -2.77 cm/s |
| Octadecenoic acid, | High | No | No | Yes | No | No | No | No | -2.19 cm/s |
| 9-Octadecenoic acid, (E) | High | No | No | Yes | No | Yes | No | No | -2.60 cm/s |
| Linoleic acid (9,12-octadecadienoic acid) | Moderate | Yes | No | Yes | No | No | No | No | -2.723 cm/s |
| Bis (2-ethylhexyl)phthalate | High | No | Yes | No | No | Yes | No | Yes | -3.39 cm/s |
| 9,12-Octadecadien-1-ol, (Z,Z) | High | Yes | No | No | No | No | No | Yes | -2.619 cm/s |
| Bis(2-ethylhexyl) terephthalate | High | No | Yes | No | No | Yes | No | Yes | -3.39 cm/s |
| 3-(6-Methyl-3-pyridyl)-1,5-diphenyl-2-pyrazoline | High | Yes | Yes | Yes | Yes | Yes | Yes | Yes | -5.12 cm/s |
