# Supplementary material for: Computational and experimental evaluation of Pisolithus arhizus metabolites targeting major efflux pumps of mastitis-associated Staphylococcus aureus
Source: PLoS One. 2026 Jul 16;21(7):e0354013. doi: 10.1371/journal.pone.0354013 (PMC13374981; doi:10.1371/journal.pone.0354013)
Supplement: S4 Table — (DOCX) [file pone.0354013.s008.docx]

**Table S4.** Molecular docking results of selected ligands of *P. arhizus's*, their binding affinity values and their bonding interactions with residues.

| Proteins | NorA | NorB | NorC | MepA |
| --- | --- | --- | --- | --- |
| PDB/Alpha fold ID | **NorA-AF-Q53459-F1-model_v4** | **AF-Q2FH03-F1-model_v6** | **7D5P** | **AF-Q2YVH4-F1-model_v6** |
| Compounds | **Binding scores (kcal mol-1)** | | | |
| N ,N Dimethylacetamide | -3.4 | -3.5 | -3.5 | -3.5 |
| m-xylene | -5.8 | -5.5 | -4.6 | -5.1 |
| o-Xylene | -5.6 | -5.1 | -4.4 | -5.0 |
| 2-Ethylhexyl acrylate | -5.5 | -5.4 | -5.2 | -5.0 |
| n-Hexadecanoic acid | -5.8 | -5.4 | -5.1 | -5.4 |
| Oleic acid (9-octadecenoic acid) | -6.4 | -5.5 | -5.4 | -5.4 |
| Octadecanoic acid | -6.5 | -5.5 | -5.6 | -5.0 |
| Linoleic acid (9,12-octadecadienoic acid) | -6.7 | -5.7 | -5.9 | -5.3 |
| 9,12-Octadecadien-1-ol, (Z,Z) | -6.3 | -5.3 | -5.1 | -5.3 |
| Bis (2-ethylhexyl)phthalate | -7.1 | -7.0 | -6.5 | -6.3 |
| Bis(2-ethylhexyl) terephthalate | -7.6 | -7.0 | -7.3 | -5.9 |
| 3-(6-Methyl-3-pyridyl)-1,5-diphenyl-2-pyrazoline | -9.3 | -8.9 | -8.4 | -8.7 |
