# Supplementary material for: Computational and experimental evaluation of Pisolithus arhizus metabolites targeting major efflux pumps of mastitis-associated Staphylococcus aureus
Source: PLoS One. 2026 Jul 16;21(7):e0354013. doi: 10.1371/journal.pone.0354013 (PMC13374981; doi:10.1371/journal.pone.0354013)
Supplement: S5 Table — (DOCX) [file pone.0354013.s009.docx]

**Table** S5**.** Molecular docking results of selected ligands of *P. arhizus*, their binding affinity values, and bonding interactions with residues

| Protein | Ligand | Interaction Type | Interacting Amino Acid Residues |
| --- | --- | --- | --- |
| MepA | **3-(6-Methyl-3-pyridyl)-1,5-diphenyl-2-pyrazoline** | van der Waals | GLY36, PHE62, MET66, PHE152, GLU156, ARG160, MET172, ASN179, THR201, SER204, ASN205 |
|  |  | Pi-Donor Hydrogen Bond | SER32, SER175 |
|  |  | Pi-Pi Stacked / T-shaped | TYR35, PHE153 |
|  |  | Alkyl / Pi-Alkyl | MET25, VAL176 |
| MepA | **Octadecanoic acid** | van der Waals | SER32, VAL33, GLY36, ASN39, ILE40, ILE55, TYR138, PHE153, VAL176, THR201, SER204, ASN205 |
|  |  | Conventional Hydrogen Bond | SER175 |
|  |  | Alkyl / Pi-Alkyl | TYR35, LEU59, PHE62, MET66, PHE152 |
|  |  | Unfavorable Donor-Donor | ASN179 |
| NorB | **3-(6-Methyl-3-pyridyl)-1,5-diphenyl-2-pyrazoline** | van der Waals | GLN31, SER147, TRP148, SER151, LEU279, ASN280, VAL282, LEU286, TYR311, PHE370, LEU374, LYS402, SER405, VAL413 |
|  |  | Pi-Sigma | ALA283 |
|  |  | Pi-Pi T-shaped | TRP27 |
|  |  | Amide-Pi Stacked | GLY409 |
|  |  | Alkyl / Pi-Alkyl | TRP27, ALA59, ALA406, ALA410 |
| NorB | **Octadecanoic acid** | van der Waals | ALA59, GLY63, VAL66, MET120, SER147, SER151, LEU279, ASN280, LEU286, TYR311, LEU374, LYS402, MET403, SER405, GLY409 |
|  |  | Conventional Hydrogen Bond | SER62 |
|  |  | Alkyl / Pi-Alkyl | PHE26, TRP27, TRP148, ALA283, ALA406 |
| NorA | **3-(6-Methyl-3-pyridyl)-1,5-diphenyl-2-pyrazoline** | van der Waals | ILE23, ARG98, PHE140, LEU218, SER219, THR223, ARG310, SER337 |
|  |  | Conventional Hydrogen Bond | GLN51 |
|  |  | Pi-Anion | GLU222 |
|  |  | Pi-Donor Hydrogen Bond | ASN340 |
|  |  | Pi-Sigma | ILE19, VAL44 |
|  |  | Pi-Pi T-shaped | PHE47 |
|  |  | Alkyl / Pi-Alkyl | PHE16, VAL22, LEU26, VAL44, PHE47, ALA48, MET109 |
| NorA | **Octadecanoic acid** | van der Waals | PHE16, LEU40, LEU43, GLN51, ARG98, GLU222, ASN340, PRO344 |
|  |  | Conventional Hydrogen Bond | SER219, THR223 |
|  |  | Alkyl / Pi-Alkyl | ILE19, VAL22, ILE23, LEU26, VAL44, PHE47, ALA48, PHE140 |
| NorC | **Octadecanoic acid** | van der Waals | TRP23, TRP139, SER140, SER143, SER147, ASN272, ASN276, VAL278, TYR307, PHE366, SER377, THR378, TYR397, LYS398 |
|  |  | Conventional Hydrogen Bond | SER401 |
|  |  | Alkyl / Pi-Alkyl | TRP144, LEU275, ALA279, LEU370, TYR373, ALA374 |
| NorC | **3-(6-Methyl-3-pyridyl)-1,5-diphenyl-2-pyrazoline** | van der Waals | TRP23, TRP139, SER143, SER147, GLY148, ASN272, ASN276, VAL278, THR281, LEU308, VAL311, PHE366, LEU370 |
|  |  | Conventional Hydrogen Bond | SER401 |
|  |  | Pi-Donor Hydrogen Bond | TYR307 |
|  |  | Pi-Sigma | ALA279 |
|  |  | Pi-Pi Stacked / T-shaped | TRP144 |
|  |  | Alkyl / Pi-Alkyl | LEU282, LYS398 |
