# Supplementary material for: Computational and experimental evaluation of Pisolithus arhizus metabolites targeting major efflux pumps of mastitis-associated Staphylococcus aureus
Source: PLoS One. 2026 Jul 16;21(7):e0354013. doi: 10.1371/journal.pone.0354013 (PMC13374981; doi:10.1371/journal.pone.0354013)
Supplement: S6 Table — (DOCX) [file pone.0354013.s010.docx]

**Table S6.**Summary of MM-GBSA binding free energy components ($\Delta G_{bind}$) calculated for the selected efflux pump–ligand complexes.

| System | ΔVDWAALS (kcal/mol) | ΔEEL (kcal/mol) | ΔEGB (kcal/mol) | ΔESURF (kcal/mol) | ΔGGAS (kcal/mol) | ΔGSOLV (kcal/mol) | ΔTOTAL (kcal/mol) |
| --- | --- | --- | --- | --- | --- | --- | --- |
| MepA – Pyrazoline | -32.09 ± 3.32 | -0.15 ± 5.04 | 21.21 ± 4.56 | -4.54 ± 0.48 | -32.25 ± 6.13 | 16.67 ± 4.38 | **-15.58 ± 3.11** |
| MepA – Octadecanoic acid | -36.11 ± 3.63 | -10.16 ± 5.11 | 16.56 ± 2.00 | -6.03 ± 0.46 | -46.27 ± 4.11 | 10.54 ± 2.03 | **-35.73 ± 3.11** |
| MepA – Tetracycline | -42.41 ± 3.22 | -12.58 ± 7.56 | 28.59 ± 6.52 | -5.48 ± 0.49 | -54.99 ± 9.00 | 23.10 ± 6.19 | **-31.89 ± 4.80** |
| NorB – Pyrazoline | -35.77 ± 2.99 | -4.50 ± 4.77 | 27.08 ± 3.59 | -4.68 ± 0.41 | -40.28 ± 6.38 | 22.40 ± 3.30 | **-17.87 ± 4.05** |
| NorB – Octadecanoic acid | -43.95 ± 6.30 | -8.24 ± 4.79 | 17.85 ± 3.64 | -6.93 ± 1.16 | -52.18 ± 9.12 | 10.92 ± 3.05 | **-41.26 ± 7.58** |
| NorB – Tetracycline | -35.35 ± 3.38 | -8.64 ± 5.39 | 29.29 ± 5.49 | -4.68 ± 0.42 | -43.99 ± 7.09 | 24.61 ± 5.35 | **-19.38 ± 4.37** |
| NorA – Pyrazoline | -39.19 ± 3.29 | 3.16 ± 3.80 | 25.15 ± 2.88 | -5.45 ± 0.37 | -36.03 ± 5.30 | 19.70 ± 2.75 | **-16.33 ± 4.31** |
| NorA – Octadecanoic acid | -36.41 ± 3.03 | -35.08 ± 6.71 | 34.37 ± 5.31 | -6.26 ± 0.31 | -71.48 ± 6.43 | 28.12 ± 5.25 | **-43.37 ± 2.76** |
| NorA – Tetracycline | -32.87 ± 3.83 | -29.05 ± 14.53 | 44.42 ± 12.10 | -4.35 ± 0.43 | -61.92 ± 13.70 | 40.06 ± 12.03 | **-21.86 ± 5.69** |
| NorC – Pyrazoline | -38.91 ± 4.12 | -19.46 ± 7.28 | 34.50 ± 4.06 | -5.07 ± 0.59 | -58.37 ± 9.56 | 29.43 ± 3.63 | **-28.94 ± 6.68** |
| NorC – Octadecanoic acid | -42.87 ± 3.41 | -6.92 ± 6.21 | 16.87 ± 2.91 | -6.64 ± 0.38 | -49.78 ± 6.67 | 10.23 ± 2.81 | **-39.55 ± 4.90** |
| NorC – Tetracycline | -35.59 ± 5.47 | -11.07 ± 6.04 | 26.35 ± 4.92 | -4.79 ± 0.69 | -46.66 ± 9.15 | 21.56 ± 4.70 | **-25.10 ± 6.73** |
