# Supplementary material for: Computational and experimental evaluation of Pisolithus arhizus metabolites targeting major efflux pumps of mastitis-associated Staphylococcus aureus
Source: PLoS One. 2026 Jul 16;21(7):e0354013. doi: 10.1371/journal.pone.0354013 (PMC13374981; doi:10.1371/journal.pone.0354013)
Supplement: Attachment — Submitted filename: Point by point response.pdf [file pone.0354013.s012.pdf]

We would like to express our sincere gratitude to the editor and reviewers for their constructive feedback and valuable suggestions. Their insightful comments have greatly contributed to improving the quality and clarity of the manuscript. We have carefully addressed all the points raised and made the necessary revisions to ensure the manuscript meets the journal's standards. Thank you for your time and effort in reviewing our work.

PONE-D-25-53988
Integrated Computational and Antimicrobial Study of Efflux Pump Inhibitors Targeting NorA in Mastitis-Associated *Staphylococcus aureus*
PLOS ONE

Dear Dr. Kifle,

Thank you for submitting your manuscript to PLOS ONE. After careful consideration, we feel that it has merit but does not fully meet PLOS ONE's publication criteria as it currently stands. Therefore, we invite you to submit a revised version of the manuscript that addresses the points raised during the review process.

Please submit your revised manuscript by Dec 28 2025 11:59PM. If you will need more time than this to complete your revisions, please reply to this message or contact the journal office at plosone@plos.org. When you're ready to submit your revision, log on to https://www.editorialmanager.com/pone/ and select the 'Submissions Needing Revision' folder to locate your manuscript file.

Please include the following items when submitting your revised manuscript:
A rebuttal letter that responds to each point raised by the academic editor and reviewer(s). You should upload this letter as a separate file labeled 'Response to Reviewers'.
A marked-up copy of your manuscript that highlights changes made to the original version. You should upload this as a separate file labeled 'Revised Manuscript with Track Changes'. An unmarked version of your revised paper without tracked changes. You should upload this as a separate file labeled 'Manuscript'.
If you would like to make changes to your financial disclosure, please include your updated statement in your cover letter. Guidelines for resubmitting your figure files are available below the reviewer comments at the end of this letter.
If applicable, we recommend that you deposit your laboratory protocols in protocols.io to enhance the reproducibility of your results. Protocols.io assigns your protocol its own identifier (DOI) so that it can be cited independently in the future. For instructions see: https://journals.plos.org/plosone/s/submission-guidelines#loc-laboratory-protocols. Additionally, PLOS ONE offers an option for publishing peer-reviewed Lab Protocol articles, which describe protocols hosted on protocols.io. Read more information on sharing protocols at https://plos.org/protocols?utm_medium=editorial-email&utm_source=authorletters&utm_campaign=protocols.

We look forward to receiving your revised manuscript.

Kind regards,

Saki Raheem, PhD
Academic Editor
PLOS ONE

Journal Requirements:
When submitting your revision, we need you to address these additional requirements.
1. Please ensure that your manuscript meets PLOS ONE's style requirements, including those for file naming. The PLOS ONE style templates can be found at https://journals.plos.org/plosone/s/file?id=wjVg/PLOSOne_formatting_sample_main_body.pdf and https://journals.plos.org/plosone/s/file?id=ba62/PLOSOne_formatting_sample_title_authors_affiliations.pdf.

Authors response: Thank you for your valuable feedback. We acknowledge the importance of adhering to the PLOS ONE style requirements. The manuscript will be carefully revised to ensure it meets all formatting guidelines, including the appropriate file naming conventions. We will make sure that the manuscript aligns with the PLOS ONE style templates, particularly those for the main body of the manuscript and the title, authors, and affiliations. The necessary adjustments will be made according to the provided formatting samples to ensure full compliance with PLOS ONE's submission standards.

2. Please include a complete copy of PLOS' questionnaire on inclusivity in global research in your revised manuscript. Our policy for research in this area aims to improve transparency in the reporting of research performed outside of researchers' own country or community. The policy applies to researchers who have travelled to a different country to conduct research, research with Indigenous populations or their lands, and research on cultural artefacts. The questionnaire can also be requested at the journal's discretion for any other submissions, even if these conditions are not met. Please find more information on the policy and a link to download a blank copy of the questionnaire here: https://journals.plos.org/plosone/s/best-practices-in-research-reporting. Please upload a completed version of your questionnaire as Supporting Information when you resubmit your manuscript.

Authors response: Thank you for your helpful comment. we have completed the Inclusivity in Global Research questionnaire as per PLOS ONE's policy and will include the completed version as Supporting Information with the resubmission of the manuscript.

3. In your Methods section, please provide additional information regarding the permits you obtained for the work. Please ensure you have included the full name of the authority that approved the field site access and, if no permits were required, a brief statement explaining why."

Authors response: Thank you for your comment. In response, I have updated the Methods section to include additional information regarding the permits obtained for the work. The fungal specimen, *Pisolithus arhizus*, was collected from privately owned land in Delay Shankai Bromikhail, Tehsil Sararogha, District South Waziristan, KPK, Pakistan, and identified by the Department of Botany, University of Sargodha. Since the material was obtained from land owned by the authors, no formal permits were required for collection. The specimen is registered under voucher number SARGU/CHEM-098.

4. Please note that PLOS One has specific guidelines on code sharing for submissions in which author-generated code underpins the findings in the manuscript. In these cases, we expect all author-generated code to be made available without restrictions upon publication of the work. Please review our guidelines at https://journals.plos.org/plosone/s/materials-and-software-sharing#loc-sharing-code and ensure that your code is shared in a way that follows best practice and facilitates reproducibility and reuse.

Authors response: Thank you for your comment regarding code sharing. After reviewing the guidelines, I would like to clarify that this study does not involve author-generated code that underpins the findings. The computational tools and software used in the research are publicly available and were appropriately referenced in the manuscript. Therefore, there is no author-generated code to submit to a repository.

5. We note that your Data Availability Statement is currently as follows: [All relevant data are within the manuscript and its Supporting Information files.]

Please confirm at this time whether or not your submission contains all raw data required to replicate the results of your study. Authors must share the "minimal data set" for their submission. PLOS defines the minimal data set to consist of the data required to replicate all study findings reported in the article, as well as related metadata and methods (https://journals.plos.org/plosone/s/data-availability#loc-minimal-data-set-definition).

For example, authors should submit the following data: - The values behind the means, standard deviations and other measures reported;

- The values used to build graphs;
- The points extracted from images for analysis.

Authors do not need to submit their entire data set if only a portion of the data was used in the reported study.

If your submission does not contain these data, please either upload them as Supporting Information files or deposit them to a stable, public repository and provide us with the relevant URLs, DOIs, or accession numbers. For a list of recommended repositories, please see https://journals.plos.org/plosone/s/recommended-repositories.

If there are ethical or legal restrictions on sharing a de-identified data set, please explain them in detail (e.g., data contain potentially sensitive information, data are owned by a third-party organization, etc.) and who has imposed them (e.g., an ethics committee). Please also provide contact information for a data access committee, ethics committee, or other institutional body to which data requests may be sent. If data are owned by a third party, please indicate how others may request data access.

Authors response: Thank you dear editor: If we refer to the raw data, the simulations performed using GROMACS generate very large files, often exceeding 2 GB per simulation. Therefore, we kindly request clarification on which specific raw data are required to fulfill the journal's "minimal data set" policy.

We are fully willing to provide the essential raw data used to generate the reported results (such as the numerical values underlying plots, extracted trajectories, and analyzed output files) as proof, either as Supporting Information or via a public repository. Please let us know precisely which components of the raw simulation data are required so that we may share the relevant files accordingly.

6. Please amend the manuscript submission data (via Edit Submission) to include author Zahida Nasreen.
Authors response: Thank you dear editor, updated and revised
7. If the reviewer comments include a recommendation to cite specific previously published works, please review and evaluate these publications to determine whether they are relevant and should be cited. There is no requirement to cite these works unless the editor has indicated otherwise.
[Note: HTML markup is below. Please do not edit.]
Authors response: Thank you dear editor for directions
Reviewers' comments:
Reviewer's Responses to Questions
Comments to the Author

1. Is the manuscript technically sound, and do the data support the conclusions?

The manuscript must describe a technically sound piece of scientific research with data that supports the conclusions. Experiments must have been conducted rigorously, with appropriate controls, replication, and sample sizes. The conclusions must be drawn appropriately based on the data presented.
Reviewer #1: Partly
Reviewer #2: Partly
2. Has the statistical analysis been performed appropriately and rigorously?
Reviewer #1: Yes
Reviewer #2: No
3. Have the authors made all data underlying the findings in their manuscript fully available?

The PLOS Data policy requires authors to make all data underlying the findings described in their manuscript fully available without restriction, with rare exception (please refer to the Data Availability Statement in the manuscript PDF file). The data should be provided as part of the manuscript or its supporting information, or deposited to a public repository. For example, in addition to summary statistics, the data points behind means, medians and variance measures should be available. If there are restrictions on publicly sharing data—e.g. participant privacy or use of data from a third party—those must be specified.
Reviewer #1: Yes
Reviewer #2: No 4. Is the manuscript presented in an intelligible fashion and written in standard English?
PLOS ONE does not copyedit accepted manuscripts, so the language in submitted articles must be clear, correct, and unambiguous. Any typographical or grammatical errors should be corrected at revision, so please note any specific errors here.
Reviewer #1: No
Reviewer #2: No

5. Reviewers' Comments to the Author

Please use the space provided to explain your answers to the questions above. You may also include additional comments for the author, including concerns about dual publication, research ethics, or publication ethics. (Please upload your review as an attachment if it exceeds 20,000 characters)

Reviewer #1:

Dear Authors

After carefully reviewing your manuscript, I can say that the topic is Ok and promising. On the other hand, I noticed the following:

1. English, in many parts especially when writing the procedures and/or preparations, is very weak. I recommend that an English native revise your manuscript. Examples: Sections 2.1 and 2.2 should be merged and rewritten, making sure that it is concise and no repetitions (repetitions appear many times in the manuscript). Section 2.4 is the same weak English! Check also section 3.3 for proper English and ters.

Authors response: We sincerely thank the reviewer for highlighting the issues regarding the English language in our manuscript. We have carefully revised the manuscript to improve clarity, grammar, and conciseness throughout.

2. In your in-text references authors mix between both the APA and the Vancouver style. Please check and correct in all places.

Authors response: We thank the reviewer for pointing out the inconsistencies in our referencing style. We have carefully reviewed all in-text citations and the reference list, and have corrected them to follow a consistent format throughout the manuscript. All references are now presented according to the journal's required style, ensuring uniformity and adherence to proper scientific referencing standards.

3. Section 2.5 in the last sentence, "The experiment was .... be repeated". This is not the reason why experiments are done in triplicates.

Authors response: We thank the reviewer for pointing out the incorrect statement in Section 2.5 regarding the use of triplicates. The sentence has been revised to accurately reflect the purpose of performing experiments in triplicates, which is to ensure reliability, reproducibility, and statistical validity of the results, rather than simply repeating the experiment.

4. Table 1: N,N-dimethylacetamides DOES NOT have a molecular formula of C6H6! Please check and correct. Additionally, 1,4-benzene dicarboxylic acid is "terephthalic acid" since we need to be consistent because you are using "phthalate" in the previous one (1,2-dicarboxylic acid).

Authors response: We thank the reviewer for pointing out the errors in Table 1. The molecular formula of N,N-dimethylacetamide has been corrected to C4H9NO. Additionally, the name 1,4-benzene dicarboxylic acid has been updated to terephthalic acid to ensure consistency.

5. Section 2.6.4. authors refer to Fig3 saying that it is a "binding interaction with the protein", while in reality in your text, this figure is missing and instead Fig 3 is the FTIR spectrum! Thus, from here on, your figures should be renumbered should you need to add the above-mentioned figure.

Authors response: We thank the reviewer for identifying the inconsistency regarding Figure 3 in Section 2.6.4. The manuscript has been corrected to ensure that the figure referred to as showing

“binding interaction with the protein” is properly included, and all subsequent figures have been renumbered accordingly to maintain consistency between the text and the figures. This ensures that all figures accurately correspond to their descriptions in the manuscript.actullay refers to target protein NorA that is Fig.1S.

6. No need to write the word “equation” after the equation, and Cl-, the minus (-) should be superscripted, while C8H10, numbers should be subscripted. Please be also consistent in style and font of the whole manuscript.

Authors response: We thank the reviewer for the valuable suggestions regarding formatting. All equations have been revised to remove the word “equation” following the expressions. Chemical notations have been corrected so that Cl^- has the minus sign as a superscript, and numbers in chemical formulas such as C_8H_{10} are properly subscripted. Additionally, we have ensured consistent style, font, and formatting throughout the manuscript to meet standard scientific presentation guidelines.

7. In the text the numbering of tables in wrong. Nothing is mentioned about Table 2, while Table 2 in text should be table 3.

Authors response: We thank the reviewer for pointing out the inconsistencies in table numbering. The manuscript has been carefully revised so that all tables are correctly numbered and properly cited in the text. Specifically, the original Table 2 has been renumbered as Table 3, and the corresponding references in the text have been updated to ensure consistency and clarity throughout the manuscript.

8. Tables 5, 6 and 7, please check the first compound for the name. Complete it please.

Authors response: We thank the reviewer for pointing out the incomplete names of the first compounds in Tables 5, 6, and 7. We have carefully reviewed all three tables and corrected the names to be complete and accurate.

9. If you use o-xylene, then why would not you use m-xylene instead of 1,3-dimethyl benzene?

Authors response: We thank the reviewer for this insightful comment. The compound 1,3-dimethylbenzene is, in fact, the systematic IUPAC name for m-xylene. To avoid confusion and ensure consistency, we have revised the manuscript to clearly indicate that 1,3-dimethylbenzene (m-xylene) is used throughout, while retaining the commonly accepted abbreviation for clarity.

10. Please check your references, None of the reference contain the "journal" name. Additionally, there are many initials in the references (e.g. 2, 4, 19, 22, ...) and there are what is written as "invalid". Please check all the reference to satisfy journal requirements.

Authors response: We thank the reviewer for highlighting the issues in the reference list. We have carefully reviewed all references and corrected them to include the full journal names, remove invalid entries, and standardize author initials according to the journal’s formatting requirements. All references have now been checked for accuracy and consistency to fully comply with the journal’s guidelines.

11. Please add also the coordinates of the town from which the fungus was collected. Where was the specimen stored? Is there a voucher number?

Authors response: We thank the reviewer for these important suggestions. We have updated the manuscript to include the geographical coordinates of the town from which the fungal specimen

was collected. Additionally, information regarding the storage of the specimen and the assigned voucher number has been added to ensure proper documentation and reproducibility of the study.

12. Though a bit mentioned in the manuscript, still the "other" interactions and/or synergistic effects of polar components of the extract (rather than the non-polar from GC analysis) should be emphasized, whether it might or might not exist (probably exists).

Authors response: We thank the reviewer for this insightful comment. We have revised the manuscript to emphasize the potential interactions and possible synergistic effects of the polar components of the extract, in addition to the non-polar compounds identified by GC analysis. While the exact nature of these interactions remains to be experimentally verified, we have highlighted their likely contribution to the observed biological activities and discussed their possible role in the overall effect of the extract.

Reviewer #2:

Thank you for giving me an opportunity to review the manuscript entitled "Integrated Computational and Antimicrobial Study of Efflux Pump Inhibitors Targeting NorA in Mastitis-Associated Staphylococcus aureus" submitted to Plos one.

1. **In abstract section introduction portion is missing.**

Authors response: Thank you for pointing this out. We have revised the abstract to include a clear introductory statement that outlines the background and rationale of the study. The updated abstract now briefly describes the significance of the problem, the scientific gap addressed, and the purpose of the investigation, ensuring a complete and coherent structure. No additional results were altered during this revision.

2. **Why authors mention about the MM-GBSA calculations results before PCA, DCCM and ADMET results?**

Response: Thank you for this valuable comment. We acknowledge the importance of maintaining a clear methodological flow in the Results section. In our study, MM-GBSA is reported before PCA, DCCM, and ADMET because it served as the initial post-docking rescoring and prioritization step. By applying MM-GBSA immediately after docking, we were able to identify the most stable and energetically favorable ligand–protein complexes that warranted more computationally intensive MD-based analyses. This sequence docking followed by MM-GBSA rescoring, then detailed MD simulation, PCA/DCCM, and finally ADMET profiling is well-established in current computational drug-discovery literature. Several studies follow this same pattern, including Jawarkar et al. (2022), who applied docking, MM-GBSA, MD simulation, and PCA; Pandey et al. (2022), who performed MM-GBSA immediately after docking before running MD and PCA; Adem et al. (2021), who used the workflow docking → MM-GBSA → MD → ADMET; and Ullah et al. (2023), who also prioritized ligands using MM-GBSA before conducting MD, PCA, and ADMET analyses. Additionally, Salmaso and Moro (2018) outline this workflow as standard practice in structure-based drug-discovery pipelines. To ensure clarity for readers, we have now added a sentence at the beginning of the post-docking section explicitly explaining the rationale for presenting MM-GBSA prior to PCA, DCCM, and ADMET.

References:

- Jawarkar, R. D., et al. (2022). *Molecules.*
- Pandey, A., et al. (2022). *Journal of Biomolecular Structure & Dynamics.*

- Adem, Ş., et al. (2021). *Journal of Molecular Graphics and Modelling.*
- Ullah, A., et al. (2023). *Computational Biology and Chemistry.*
- Salmaso, V., & Moro, S. (2018). *Frontiers in Pharmacology*.

3. **Check the line "2.2 Preparation and extraction of Fungi metabolites"**

Authors response: We thank the reviewer for pointing this out. The section titled "2.2 Preparation and extraction of Fungi metabolites" has now been carefully reviewed and corrected for clarity, accuracy, and consistency. We have refined the wording, ensured proper formatting, and corrected any grammatical or typographical errors present in the original version. The updated section is now clearer and more precise in describing the methodology.

4. **Concentration of methanol should mention during extraction of active compounds in methodology section.**

Authors response: We thank the reviewer for this suggestion. The methodology section (2.2 Preparation and Extraction of Fungal Metabolites) has been revised to specify that 80% methanol was used for the extraction of active compounds, ensuring clarity and reproducibility of the procedure.

5. **In introduction section, 1st paragraph should me write in one or two sentences (write within 25-35 words).**

Authors response: We thank the reviewer for this suggestion. The first paragraph of the Introduction has been revised to a concise version of 32 words, summarizing the background and significance of the study while maintaining clarity and focus.

6. **Why authors targeted only norA why not others (norB, norC, mepA and others)?**

Authors response: Thank you for your insightful question. We specifically targeted NorA in this study due to its well-established role in fluoroquinolone and multidrug resistance in Staphylococcus aureus, particularly in bovine mastitis. NorA is one of the most extensively studied efflux pumps in S. aureus, and its inhibition has shown promising potential to reverse antimicrobial resistance. While other efflux pumps such as NorB, NorC, and MepA are also important in antimicrobial resistance, they are less characterized in the context of S. aureus strains associated with bovine mastitis, and NorA has been identified as a key player in resistance mechanisms for this condition. We agree that investigating these other pumps in future studies would be valuable, and we plan to explore this in subsequent research.

7. **Rewrite the line 2.6.2 section "Molecular weight, lipophilicity or hydrophilicity, gastrointestinal absorption, blood– brain barrier (BBB) permeability, p-glycoprotein substrate, skin permeability, toxicity, drug likelihood, and bioavailability score were among the data collected and evaluated from the instruments"**

Authors response: Thank you for your comment. As per your suggestion, we rewrite it in the manuscript.

8. **In case of Antibacterial assay how many sets were taken?**

Authors response: We thank the reviewer for this observation. In the antibacterial assay, all experiments were performed in triplicate (three independent sets) to ensure accuracy, reproducibility, and statistical reliability of the results.

9. **Authors should Transfer Table 1, 5, 6 and Fig. 2, 5 as supplementary.**

Authors response: Thanks a lot for your valuable comment. We have transferred the tables 1,5,6, and figure 2,5 into supplementary file, and add their number as 1S, 5S, 6Setc in manuscript as well as we also rearrange the numbering of tables and figures in whole manuscript.

10. **Authors should split a single sentence into two or more simpler sentences which lead to enhanced readability.**

Authors response: We thank the reviewer for this valuable suggestion. We have carefully reviewed the manuscript and split long, complex sentences into two or more simpler sentences throughout the text, improving clarity and overall readability.

11. Authors should check grammatical and linguistic errors throughout the manuscript.

Authors response: We thank the reviewer for this important observation. The manuscript has been thoroughly reviewed and carefully edited to correct grammatical, linguistic, and typographical errors, ensuring improved clarity, readability, and overall quality of the text.

12. **Check the references properly "3.3 Antibacterial Activity".**

Authors response: We thank the reviewer for pointing this out. All references cited in section 3.3 Antibacterial Activity have been carefully checked and corrected for accuracy, consistency, and proper formatting according to the journal's guidelines.

13. **Authors should perform in vitro validation of efflux pumps inhibitory effect of 3-(6-Methyl-3-pyridyl)-1,5-diphenyl-2-pyrazoline and octadecanoic acid**.

Authors response: We thank the reviewer for this valuable suggestion. Due to resource and time limitations, we were unable to perform in vitro validation of the efflux pump inhibitory activity in the current study. We have acknowledged this limitation in the manuscript and recommended it as a potential direction for future research.

Comments to editor: Thank you for your valuable feedback and the opportunity to revise our manuscript. We have carefully addressed all of the editor's and reviewers' comments and have made the necessary revisions to improve the manuscript.

In response to the reviewer comments, we have highlighted all changes and responses in red text to indicate where revisions were made and to clearly present our responses. Additionally, all grammatical improvements have been marked in blue text to reflect the changes made for clarity and readability.

We trust that the revisions meet the expectations of the journal and are grateful for the time and effort put into reviewing our work.
