# Supplementary material for: Computational and experimental evaluation of Pisolithus arhizus metabolites targeting major efflux pumps of mastitis-associated Staphylococcus aureus
Source: PLoS One. 2026 Jul 16;21(7):e0354013. doi: 10.1371/journal.pone.0354013 (PMC13374981; doi:10.1371/journal.pone.0354013)
Supplement: Attachment — Submitted filename: point to point responses R4.pdf [file pone.0354013.s014.pdf]

Point by Point Response

The authors would like to sincerely thank the Editor and the Reviewers for their careful evaluation of our manuscript and for their valuable comments and constructive suggestions. Their insightful feedback has significantly improved the quality, clarity, and scientific rigor of the manuscript. All comments have been carefully considered, and the manuscript has been revised accordingly. We hope that the revisions satisfactorily address all concerns raised by the reviewers and editor.

Reviewer #3:

Reviewer #3: The manuscript has improved substantially compared with the previous version, but important issues remain regarding:

- Overinterpretation of efflux pump inhibition,

- persistent language problems,

- incomplete integration of NorC analyses,

- and excessive speculative discussion.

A further moderate revision focused on scientific rigor, consistency, and language quality is still recommended. More specifically:

Author response: We thank the reviewer for this valuable comment and agree that the experimental data presented in the current study do not directly demonstrate efflux pump inhibition. While our computational analyses evaluated the interactions of *Pisolithus arhizus* metabolites with major mastitis-associated *Staphylococcus aureus* efflux pumps, and the experimental studies confirmed antibacterial activity, no dedicated efflux pump inhibition assays (e.g., ethidium bromide accumulation assays, efflux inhibition assays, antibiotic synergism studies, or transporter-specific validation experiments) were performed.

In response, we have carefully revised the manuscript to avoid overstating the evidence. Statements implying direct confirmation of efflux pump inhibition have been moderated throughout the Abstract, Results, Discussion, and Conclusions sections. The observed antibacterial activity is now discussed independently, whereas the involvement of efflux pump inhibition is presented as a hypothesis supported by computational predictions that requires further experimental validation.

To further address this concern, the title has been revised to better reflect the scope of the study and the nature of the evidence presented. We have also added a limitation statement emphasizing that future studies employing specific efflux pump-related assays will be necessary to confirm the proposed mechanism of action.

These revisions ensure that the conclusions are fully supported by the available computational and experimental data.

1. The manuscript still overstates the evidence for efflux pump inhibition. The additional experiments presented only demonstrate antibacterial activity and do not directly validate efflux pump inhibition. Statements linking antibacterial

effects to efflux inhibition therefore remain speculative and should be moderated throughout the manuscript unless supported by dedicated efflux-related assays such as ethidium bromide accumulation assay, efflux inhibition assay, synergism assay with antibiotics, or transporter-specific validation

Author response: We thank the reviewer for this important comment. We agree that the experimental assays performed in this study demonstrate antibacterial activity rather than direct efflux pump inhibition. To address this concern, we carefully revised the manuscript to moderate statements that could be interpreted as definitive evidence of efflux pump inhibition.

2. Despite the authors' claim of extensive language revision, numerous grammatical, stylistic, and sentence-construction issues persist across the manuscript. Several expressions remain awkward or scientifically imprecise, indicating that the manuscript still requires thorough professional English editing before publication.

Author response: We thank the reviewer for this observation. We agree that language clarity is essential for effective scientific communication. In response, the manuscript has undergone an additional comprehensive round of English-language editing to improve grammar, sentence structure, scientific phrasing, readability, and overall consistency. Numerous awkward and repetitive expressions were revised, and several sections were rewritten for greater clarity and precision. We have carefully reviewed the entire manuscript to ensure that the language now meets the standards expected for publication.

3. There is also inconsistency regarding the inclusion of the NorC target in the computational analyses. While the authors state that NorC was incorporated comprehensively, several downstream analyses, figures, and tables appear to include only NorA, NorB, and MepA. This discrepancy should be clarified and corrected for consistency.

Author response: We thank the reviewer for carefully identifying this inconsistency regarding the inclusion of the NorC efflux pump in the computational analyses.

Upon re-examination of the manuscript, we found that although NorC was included in the initial computational workflow, its representation was inadvertently omitted or inconsistently presented in certain downstream analyses, figures, and tables. We agree that this inconsistency could lead to confusion regarding the scope of the study.

To address this issue, we have thoroughly reviewed all sections of the manuscript and implemented the necessary corrections. Specifically, we have ensured that the description of the computational analyses accurately reflects the efflux pump targets included in the study. The relevant figures, tables, supplementary materials, and corresponding text have been revised to consistently indicate whether NorC was included in each analysis. Where NorC data were available, the corresponding results have been incorporated. Where NorC was not included in a particular downstream analysis, this has been explicitly stated and justified in the revised manuscript.

These revisions eliminate the discrepancy between the methodology and the reported results and provide a consistent and transparent presentation of the computational evaluation of NorA, NorB, NorC, and MepA efflux pumps throughout the manuscript.

We appreciate the reviewer's observation, which helped improve the accuracy and consistency of the manuscript.

4. The revised manuscript remains excessively long and repetitive, particularly in the Results and Discussion sections. Similar explanations of molecular interactions and antibacterial relevance are repeated multiple times, and the text would benefit from substantial condensation to improve clarity and readability.

Author response: We thank the reviewer for this constructive suggestion regarding the length and organization of the manuscript. We agree that portions of the Results and Discussion sections contained repetitive descriptions, particularly concerning molecular interactions, docking outcomes, and their potential antibacterial implications.

In response, we have carefully revised these sections to improve conciseness, clarity, and readability. Repetitive explanations of ligand–protein interactions, binding patterns, and antibacterial relevance have been consolidated, while redundant descriptions across different target proteins have been removed. Similar findings are now discussed collectively where appropriate, rather than being described repeatedly for each target.

Additionally, several paragraphs have been shortened or restructured to emphasize the most significant findings and their biological relevance. The Discussion section has also been streamlined to focus on the interpretation of key results and their implications, while avoiding unnecessary repetition of information already presented in the Results section.

These revisions have substantially reduced the overall length of the manuscript and improved the logical flow of the text, resulting in a clearer and more reader-friendly presentation of the study. We appreciate the reviewer's comment, which helped us enhance the quality and readability of the manuscript.

5. Although the authors acknowledged that the pyrazoline compound identification remains tentative based on GC–MS library matching, the manuscript still discusses the compound in several sections as though it were definitively identified and biologically confirmed. The tentative nature of the identification should be consistently reflected throughout the text.

Author response: We thank the reviewer for this important observation. We agree that the identification of the pyrazoline compound in the present study is tentative and based solely on GC–MS spectral matching against reference library databases. As no authentic standard, additional spectroscopic characterization (e.g., NMR, FTIR, or LC–MS/MS), or compound isolation was performed, definitive structural confirmation cannot be claimed.

In response to the reviewer's comment, we have carefully revised the manuscript to ensure that the tentative nature of the compound identification is consistently reflected throughout the text. Statements that previously referred to the pyrazoline compound as a definitively identified or biologically confirmed metabolite have been modified to indicate that it is a **putatively identified**, **tentatively assigned**, or **GC–MS library-matched** compound.

Furthermore, interpretations regarding its biological activity have been appropriately moderated. The computational and antibacterial findings are now discussed as being associated with the putatively identified pyrazoline-like compound, rather than as confirmation of the activity of a definitively characterized molecule. We have also emphasized in the Discussion and Limitations sections that further purification, structural elucidation, and experimental validation are required to confirm the identity and biological properties of this compound.

These revisions have been implemented consistently throughout the Abstract, Results, Discussion, Conclusions, figures, tables, and supplementary materials to ensure that the manuscript accurately reflects the level of evidence provided by the current study.

6. Some methodological and statistical details remain insufficiently reported or inconsistently formatted. These include unclear presentation of CFU values, limited reporting of statistical analyses and p-values, and inadequate distinction between biological and technical replicates.

Author response: We thank the reviewer for highlighting the need for greater clarity and consistency in the reporting of methodological and statistical details. We agree that transparent reporting of experimental design and statistical analyses is essential for the reproducibility and interpretation of the study.

In response, we have carefully reviewed and revised the Materials and Methods, Results, tables, and figure legends to improve the presentation of methodological and statistical information throughout the manuscript.

Specifically:

1. **CFU Reporting:** Colony-forming unit (CFU) data have been reformatted and presented consistently throughout the manuscript using appropriate scientific notation and standardized units (e.g., CFU/mL). Any ambiguous or inconsistently reported values have been corrected.
2. **Statistical Analyses:** The statistical methods used for data analysis have been described in greater detail, including the statistical tests employed, significance thresholds, software used for analysis, and the criteria for determining statistical significance. Where applicable, corresponding p-values have been added to the Results section, tables, and figure legends.
3. **Biological and Technical Replicates:** The experimental design has been clarified by explicitly distinguishing between biological replicates and technical replicates. The number of replicates performed for each experiment is now clearly stated in the Materials and Methods section and, where appropriate, in the relevant figure legends and table footnotes.
4. **Data Presentation:** Measures of variability (e.g., mean ± standard deviation or standard error, as appropriate) have been reported consistently across all datasets, and statistical annotations have been standardized throughout the manuscript.

These revisions were undertaken to improve transparency, reproducibility, and consistency in the reporting of the experimental and statistical procedures. We appreciate the reviewer's comment, which has helped strengthen the methodological rigor and overall quality of the manuscript.

7. Finally, several scientific interpretations remain overly speculative and extend beyond the experimental evidence provided. Claims related to membrane targeting, wound healing relevance, therapeutic applications, and broad-spectrum efflux inhibition should be tempered to better align with the data generated in the study.

Author response: We thank the reviewer for this valuable comment and agree that several interpretations in the previous version of the manuscript extended beyond the direct evidence generated in the present study.

In response, we have carefully reviewed the manuscript and revised statements that could be interpreted as speculative or insufficiently supported by the available data.

We appreciate the reviewer's observation, which has helped us improve the scientific rigor, balance, and accuracy of the manuscript.

Comments to editor:

We respectfully inform the Editor that all reviewer comments have been carefully addressed in the revised manuscript. For clarity, responses to Reviewer 3 comments are **highlighted in red,** and grammatical improvements are **marked in Blue**.
