# Supplementary material for: Computational and experimental evaluation of Pisolithus arhizus metabolites targeting major efflux pumps of mastitis-associated Staphylococcus aureus
Source: PLoS One. 2026 Jul 16;21(7):e0354013. doi: 10.1371/journal.pone.0354013 (PMC13374981; doi:10.1371/journal.pone.0354013)
Supplement: Attachment — Submitted filename: Point by point response final.docx [file pone.0354013.s013.docx]

**Acknowledgments**

We would like to express our sincere gratitude to the editor and reviewers for their constructive comments and valuable suggestions. Their insights significantly contributed to improving the quality and clarity of this manuscript.

We are especially grateful to Reviewer #2 for suggesting the inclusion of additional efflux pump targets (NorB, NorC, and MepA), which enhanced the scientific value of the study. We also appreciate their recommendation to perform in vitro assays, which we have now incorporated into the manuscript, providing comprehensive experimental validation of our findings.

We thank Reviewer #3 for their thorough review and suggestions for enhancing the manuscript's readability and consistency, especially regarding language editing, the reference list, and compound nomenclature. These revisions have been carefully implemented to ensure the manuscript meets the journal's standards.

Once again, our sincere thanks to all for their time and effort in reviewing the manuscript.

**RESPONSE TO REVIEWERS COMMENTS**

**Reviewer #1**

**Comment:** “Dear Authors

Thanks for correcting the manuscript.”

**Authors Response:** We are grateful for your response respected reviewer.

**Reviewer #2**

**Comment 1:** The authors are encouraged to target norB, norC, and mepA, which are also key contributors to antimicrobial resistance in Staphylococcus aureus. Their inclusion in the results would likely enhance the scientific value of the study.

**Authors Response:** Thanks a lot dear reviewer for suggestion. Now we have also added NorB, NorC, and mepA, targets in our manuscript as per your suggestion.

**Comment 2:** The authors should carry out in vitro assays to validate the inhibitory effects of 3-(6-methyl-3-pyridyl)-1,5-diphenyl-2-pyrazoline and octadecanoic acid on bacterial efflux pumps.

https://doi.org/10.3390/ph18030323

https://doi.org/10.1080/07391102.2021.1976277

https://doi.org/10.1016/j.prenap.2024.100070

https://doi.org/10.1007/s00284-024-03836-0

<https://doi.org/10.3389/fmicb.2018.02990>

**Authors Response:** We sincerely appreciate the reviewer’s suggestion to validate the inhibitory effects of 3-(6‑methyl‑3‑pyridyl)‑1,5‑diphenyl‑2‑pyrazoline and octadecanoic acid on bacterial efflux pumps through in vitro assays. In the revised manuscript, we have incorporated a comprehensive experimental evaluation that includes both well diffusion antibacterial assays and Minimum Inhibitory Concentration (MIC) determinations. Additionally, we have discussed these in vitro results in the context of efflux pump inhibition and supported our interpretations with relevant literature.

**Reviewer #3**

**Comment 1:** The manuscript still requires substantial professional English language editing. Numerous grammatical errors, awkward phrases, and overly long or broken sentences remain throughout the text, despite the authors’ claim of thorough revision. A careful, line-by-line edit by a native English speaker or professional editing service is necessary to improve clarity and readability.

**Authors Response:** Thank you for this important comment. We performed a comprehensive, line-by-line English language edit across the entire manuscript to improve clarity, grammar, and readability. Specifically, we corrected grammatical and typographical errors, rephrased awkward constructions, reduced redundancy, and split overly long sentences. We also standardized technical terminology and abbreviations throughout the text to ensure consistency. These revisions have been implemented throughout the manuscript.

**Comment 2:** The reference list is incomplete and inconsistently formatted. Several entries lack essential elements such as journal names, full author details, and page numbers. The entire reference section must be reconstructed according to a consistent standard style, following the journal guidelines, ensuring that all citations are complete and properly punctuated.

**Authors** Response: Thanks a lot dear reviewer for pointing out. We have now revised the whole manuscript as per your suggestions,

**Comment 3:** There is inconsistency between the response letter and the revised manuscript. Some claimed revisions are not fully reflected in the clean version of the manuscript, and discrepancies remain between the tracked-changes version and the final submitted file. The authors should carefully reconcile both versions to ensure accuracy and completeness of all revisions.

**Authors Response:** We thank the reviewer for pointing this out. We have carefully double-checked and reconciled the tracked-changes version with the clean version of the manuscript to ensure that all revisions described in the response letter are accurately reflected. All updates, including changes to compound nomenclature, GC–MS justification, extract preparation, antibacterial assays, MIC values, and formatting corrections, have now been thoroughly incorporated to ensure consistency and completeness.

**Comment 4:** The concentration of methanol used for extraction is missing in the Methods section. Although 80% methanol is mentioned in the response letter, this detail does not appear in the manuscript. The exact concentration must be clearly stated to ensure reproducibility.

**Authors Response:** Thank you dear reviwer for pointing this out. The Methods section has been revised to explicitly state the solvent composition used for extraction. Specifically, the extract was prepared using 80% (v/v) methanol (methanol:water, 80:20) (Section 2.1).

**Comment 5:** Compound nomenclature is inconsistent across the manuscript. In particular, the phthalate compound is referred to by different names in the text and tables. A single, consistent chemical name should be used throughout all sections, tables, and figures.

**Authors Response:** We thank the reviewer for pointing out the inconsistencies in compound nomenclature throughout the manuscript. We carefully reviewed the entire text, tables, figures, and supplementary materials, and standardized the naming of all chemical compounds to ensure clarity and reproducibility.

**Comment 6:** Chemical formulas are not consistently formatted with proper subscripts and superscripts. A final technical check is required to ensure that all molecular formulas are correctly formatted in the text, tables, and figures.

**Authors Response:** We thank the reviewer for highlighting the inconsistency in the formatting of chemical formulas throughout the manuscript. We have carefully reviewed all text, tables, figures, and supplementary materials, and corrected the formatting to ensure that all molecular formulas display proper subscripts and superscripts according to standard chemical notation.

**Comment 7:** The preparation of the extract solution used for bioassays is insufficiently described. The manuscript should clearly detail how the extract was dissolved, diluted, and prepared prior to antimicrobial testing to ensure methodological transparency.

**Authors Response:** We thank the reviewer for pointing out the need for greater detail regarding the preparation of the Pisolithus arhizus extract solution for bioassays. In the revised manuscript, we have clarified the procedure to ensure methodological transparency and reproducibility. The Materials and Methods section now specifies how the methanolic extract was dissolved, diluted, and prepared prior to antimicrobial testing.

**Comment 8:** Minimum inhibitory concentration (MIC) values are not reported. These values should be provided to properly support and quantify the antibacterial activity claims. The current disk diffusion assay is limited.

**Authors Response:** We thank the reviewer for emphasizing the importance of reporting Minimum Inhibitory Concentration (MIC) values to support and quantify the antibacterial activity. In the revised manuscript, we have now included MIC determinations for all tested samples.

**Comment 9:** A full description of bacterial features should be reported, including the source/origin and resistance profile. Additionally, the reference strains should be included to ensure methodological reproducibility.

**Authors Response:** We thank the reviewer for pointing out; The need for a full description of the bacterial strain, including its source, reference number, and resistance profile, to ensure reproducibility of the antibacterial assays. In the revised manuscript, we have updated the Materials and Methods section to provide this information in detail.

**Comment 10:** A validation of computational results would add a significant value to the study.

**Authors Response:** We thank the reviewer for highlighting the importance of validating the computational predictions. In the revised manuscript, we have incorporated in vitro antibacterial and MIC assays to support the in silico findings. Specifically, 3-(6-methyl-3-pyridyl)-1,5-diphenyl-2-pyrazoline (pyrazoline) and octadecanoic acid, which were predicted via molecular docking and MM-GBSA analyses to interact strongly with Staphylococcus aureus efflux pumps, were tested for antibacterial activity using well diffusion and Minimum Inhibitory Concentration (MIC) assays against S. aureus (ATCC 23235).

**Comment 11:** The justification for using GC–MS analysis on a methanol extract is unclear. Since GC–MS is typically suited for volatile and semi-volatile compounds, the authors should justify its appropriateness for this extract or clarify any derivatization or methodological considerations.

**Authors Response:** We thank the reviewer for the comment. We have updated the manuscript to clarify that GC–MS was used because the Pisolithus arhizus methanolic extract contains volatile and semi-volatile metabolites (fatty acids, phthalates, xylene derivatives, and pyrazoline derivatives) suitable for direct GC–MS analysis. No derivatization was required, as the compounds were sufficiently volatile and thermally stable.

**Comment 12:** The manuscript is excessively long, particularly in the Results and Discussion sections. Redundant explanations and overly detailed analysis should be reduced to improve focus, clarity, and overall readability.

**Authors Response:** Thanks a lot dear reviewer for your comment. Now we have updated the manuscript.

**Comments to editor:**

**Dear Editor’s,**

We have carefully addressed all the comments and suggestions provided. The revisions have been made accordingly, with changes **highlighted in red text** and grammatical improvements **marked in blue text**. We believe these revisions have strengthened the manuscript, and we hope it now meets the journal's standards. Thank you for your time and consideration.
