# Supplementary material for: Computational and experimental evaluation of Pisolithus arhizus metabolites targeting major efflux pumps of mastitis-associated Staphylococcus aureus
Source: PLoS One. 2026 Jul 16;21(7):e0354013. doi: 10.1371/journal.pone.0354013 (PMC13374981; doi:10.1371/journal.pone.0354013)
Supplement: Peer review history

# Peer review: Decision Letter 0

**DOI:** 10.1371/journal.pone.0354013.r001

**Authors:** Saki Raheem

13 Nov 2025

PONE-D-25-53988

Integrated Computational and Antimicrobial Study of Efflux Pump Inhibitors Targeting NorA in Mastitis-Associated Staphylococcus aureus

PLOS ONE

Dear Dr. Kifle,

Thank you for submitting your manuscript to PLOS ONE. After careful consideration, we feel that it has merit but does not fully meet PLOS ONE’s publication criteria as it currently stands. Therefore, we invite you to submit a revised version of the manuscript that addresses the points raised during the review process.

Please submit your revised manuscript by Dec 28 2025 11:59PM. If you will need more time than this to complete your revisions, please reply to this message or contact the journal office at plosone@plos.org. When you're ready to submit your revision, log on to https://www.editorialmanager.com/pone/ and select the 'Submissions Needing Revision' folder to locate your manuscript file.

Please include the following items when submitting your revised manuscript:

- A rebuttal letter that responds to each point raised by the academic editor and reviewer(s). You should upload this letter as a separate file labeled 'Response to Reviewers'.
- A marked-up copy of your manuscript that highlights changes made to the original version. You should upload this as a separate file labeled 'Revised Manuscript with Track Changes'.
- An unmarked version of your revised paper without tracked changes. You should upload this as a separate file labeled 'Manuscript'.

If you would like to make changes to your financial disclosure, please include your updated statement in your cover letter. Guidelines for resubmitting your figure files are available below the reviewer comments at the end of this letter.

If applicable, we recommend that you deposit your laboratory protocols in protocols.io to enhance the reproducibility of your results. Protocols.io assigns your protocol its own identifier (DOI) so that it can be cited independently in the future. For instructions see: https://journals.plos.org/plosone/s/submission-guidelines#loc-laboratory-protocols. Additionally, PLOS ONE offers an option for publishing peer-reviewed Lab Protocol articles, which describe protocols hosted on protocols.io. Read more information on sharing protocols at https://plos.org/protocols?utm_medium=editorial-email&utm_source=authorletters&utm_campaign=protocols.

We look forward to receiving your revised manuscript.

Kind regards,

Saki Raheem, PhD

Academic Editor

PLOS ONE

Journal Requirements:

When submitting your revision, we need you to address these additional requirements.

1. Please ensure that your manuscript meets PLOS ONE's style requirements, including those for file naming. The PLOS ONE style templates can be found at

https://journals.plos.org/plosone/s/file?id=wjVg/PLOSOne_formatting_sample_main_body.pdf and

https://journals.plos.org/plosone/s/file?id=ba62/PLOSOne_formatting_sample_title_authors_affiliations.pdf.

2. Please include a complete copy of PLOS’ questionnaire on inclusivity in global research in your revised manuscript. Our policy for research in this area aims to improve transparency in the reporting of research performed outside of researchers’ own country or community. The policy applies to researchers who have travelled to a different country to conduct research, research with Indigenous populations or their lands, and research on cultural artefacts. The questionnaire can also be requested at the journal’s discretion for any other submissions, even if these conditions are not met.  Please find more information on the policy and a link to download a blank copy of the questionnaire here: https://journals.plos.org/plosone/s/best-practices-in-research-reporting. Please upload a completed version of your questionnaire as Supporting Information when you resubmit your manuscript.

3. In your Methods section, please provide additional information regarding the permits you obtained for the work. Please ensure you have included the full name of the authority that approved the field site access and, if no permits were required, a brief statement explaining why."

4. Please note that PLOS One has specific guidelines on code sharing for submissions in which author-generated code underpins the findings in the manuscript. In these cases, we expect all author-generated code to be made available without restrictions upon publication of the work. Please review our guidelines at https://journals.plos.org/plosone/s/materials-and-software-sharing#loc-sharing-code and ensure that your code is shared in a way that follows best practice and facilitates reproducibility and reuse.

5. We note that your Data Availability Statement is currently as follows: [All relevant data are within the manuscript and its Supporting Information files.]

Please confirm at this time whether or not your submission contains all raw data required to replicate the results of your study. Authors must share the “minimal data set” for their submission. PLOS defines the minimal data set to consist of the data required to replicate all study findings reported in the article, as well as related metadata and methods (https://journals.plos.org/plosone/s/data-availability#loc-minimal-data-set-definition).

For example, authors should submit the following data:

- The values behind the means, standard deviations and other measures reported;

- The values used to build graphs;

- The points extracted from images for analysis.

Authors do not need to submit their entire data set if only a portion of the data was used in the reported study.

If your submission does not contain these data, please either upload them as Supporting Information files or deposit them to a stable, public repository and provide us with the relevant URLs, DOIs, or accession numbers. For a list of recommended repositories, please see https://journals.plos.org/plosone/s/recommended-repositories.

If there are ethical or legal restrictions on sharing a de-identified data set, please explain them in detail (e.g., data contain potentially sensitive information, data are owned by a third-party organization, etc.) and who has imposed them (e.g., an ethics committee). Please also provide contact information for a data access committee, ethics committee, or other institutional body to which data requests may be sent. If data are owned by a third party, please indicate how others may request data access.

6. Please amend the manuscript submission data (via Edit Submission) to include author Zahida Nasreen.

7. If the reviewer comments include a recommendation to cite specific previously published works, please review and evaluate these publications to determine whether they are relevant and should be cited. There is no requirement to cite these works unless the editor has indicated otherwise.

[Note: HTML markup is below. Please do not edit.]

Reviewers' comments:

Reviewer's Responses to Questions

**Comments to the Author**

1. Is the manuscript technically sound, and do the data support the conclusions?

The manuscript must describe a technically sound piece of scientific research with data that supports the conclusions. Experiments must have been conducted rigorously, with appropriate controls, replication, and sample sizes. The conclusions must be drawn appropriately based on the data presented.

Reviewer #1: Partly

Reviewer #2: Partly

**********

2. Has the statistical analysis been performed appropriately and rigorously?

Reviewer #1: Yes

Reviewer #2: No

**********

3. Have the authors made all data underlying the findings in their manuscript fully available?

The PLOS Data policy requires authors to make all data underlying the findings described in their manuscript fully available without restriction, with rare exception (please refer to the Data Availability Statement in the manuscript PDF file). The data should be provided as part of the manuscript or its supporting information, or deposited to a public repository. For example, in addition to summary statistics, the data points behind means, medians and variance measures should be available. If there are restrictions on publicly sharing data—e.g. participant privacy or use of data from a third party—those must be specified.

Reviewer #1: Yes

Reviewer #2: No

**********

4. Is the manuscript presented in an intelligible fashion and written in standard English?

PLOS ONE does not copyedit accepted manuscripts, so the language in submitted articles must be clear, correct, and unambiguous. Any typographical or grammatical errors should be corrected at revision, so please note any specific errors here.

Reviewer #1: No

Reviewer #2: No

**********

5. Review Comments to the Author

Please use the space provided to explain your answers to the questions above. You may also include additional comments for the author, including concerns about dual publication, research ethics, or publication ethics. (Please upload your review as an attachment if it exceeds 20,000 characters)

Reviewer #1: Dear Authors

After carefully reviewing your manuscript, I can say that the topic is Ok and promising. On the other hand, I noticed the following:

1. English, in many parts especially when writing the procedures and/or preparations, is very weak. I recommend that an English native revise your manuscript. Examples: Sections 2.1 and 2.2 should be merged and rewritten, making sure that it is concise and no repetitions (repetitions appear many times in the manuscript). Section 2.4 is the same weak English! Check also section 3.3 for proper English and ters.

2. In your in-text references authors mix between both the APA and the Vancouver style. Please check and correct in all places.

3. Section 2.5 in the last sentence, "The experiment was .... be repeated". This is not the reason why experiments are done in triplicates.

4. Table 1: N,N-dimethylacetamides DOES NOT have a molecular formula of C6H6! Please check and correct. Additionally, 1,4-benzene dicarboxylic acid is "terephthalic acid" since we need to be consistent because you are using "phthalate" in the previous one (1,2-dicarboxylic acid).

5. Section 2.6.4. authors refer to Fig3 saying that it is a "binding interaction with the protein", while in reality in your text, this figure is missing and instead Fig 3 is the FTIR spectrum! Thus, from here on, your figures should be renumbered should you need to add the above-mentioned figure.

6. No need to write the word "equation" after the equation, and Cl-, the minus (-) should be superscripted, while C8H10, numbers should be subscripted. Please be also consistent in style and font of the whole manuscript.

7. In the text the numbering of tables in wrong. Nothing is mentioned about Table 2, while Table 2 in text should be table 3.

8. Tables 5, 6 and 7, please check the first compound for the name. Complete it please.

9. If you use o-xylene, then why would not you use m-xylene instead of 1,3-dimethyl benzene?

10. Please check your references, None of the reference contain the "journal" name. Additionally, there are many initials in the references (e.g. 2, 4, 19, 22, ...) and there are what is written as "invalid". Please check all the reference to satisfy journal requirements.

11. Please add also the coordinates of the town from which the fungus was collected. Where was the specimen stored? Is there a voucher number?

12. Though a bit mentioned in the manuscript, still the "other" interactions and/or synergistic effects of polar components of the extract (rather than the non-polar from GC analysis) should be emphasized, whether it might or might not exist (probably exists).

Generally, procedures are good, but in some instances, more details are needed.

All the best.

Reviewer #2: Thank you for giving me an opportunity to review the manuscript entitled “Integrated Computational and Antimicrobial Study of Efflux Pump Inhibitors Targeting NorA in Mastitis-Associated Staphylococcus aureus” submitted to Plos one.

• In abstract section introduction portion is missing.

• Why authors mention about the MM-GBSA calculations results before PCA, DCCM and ADMET results?

• Check the line “2.2 Preparation and extraction of Fungi metabolites”

• Concentration of methanol should mention during extraction of active compounds in methodology section.

• In introduction section, 1st paragraph should me write in one or two sentences (write within 25-35 words).

• Why authors targeted only norA why not others (norB, norC, mepA and others)?

• Rewrite the line 2.6.2 section “Molecular weight, lipophilicity or hydrophilicity, gastrointestinal absorption, blood–

brain barrier (BBB) permeability, p-glycoprotein substrate, skin permeability, toxicity, drug likelihood, and

bioavailability score were among the data collected and evaluated from the instruments”

• In case of Antibacterial assay how many sets were taken?

• Authors should Transfer Table 1, 5, 6 and Fig. 2, 5 as supplementary.

• Authors should split a single sentence into two or more simpler sentences which lead to enhanced readability.

• Authors should check grammatical and linguistic errors throughout the manuscript.

• Check the references properly “3.3 Antibacterial Activity”.

• Authors should perform in vitro validation of efflux pumps inhibitory effect of 3-(6- Methyl-3-pyridyl)-1,5-

diphenyl-2-pyrazoline and octadecanoic acid.

**********

6. PLOS authors have the option to publish the peer review history of their article (what does this mean?). If published, this will include your full peer review and any attached files.

If you choose “no”, your identity will remain anonymous but your review may still be made public.

**Do you want your identity to be public for this peer review?**  For information about this choice, including consent withdrawal, please see our Privacy Policy.

Reviewer #1: **Yes:** Michel Hanania

Reviewer #2: No

**********

[NOTE: If reviewer comments were submitted as an attachment file, they will be attached to this email and accessible via the submission site. Please log into your account, locate the manuscript record, and check for the action link "View Attachments". If this link does not appear, there are no attachment files.]

To ensure your figures meet our technical requirements, please review our figure guidelines: https://journals.plos.org/plosone/s/figures

You may also use PLOS’s free figure tool, NAAS, to help you prepare publication quality figures: https://journals.plos.org/plosone/s/figures#loc-tools-for-figure-preparation.

NAAS will assess whether your figures meet our technical requirements by comparing each figure against our figure specifications.

# Author comment: Author response to Decision Letter 1

**DOI:** 10.1371/journal.pone.0354013.r002

5 Jan 2026

We would like to express our sincere gratitude to the editor and reviewers for their

constructive feedback and valuable suggestions. Their insightful comments have

greatly contributed to improving the quality and clarity of the manuscript. We have

carefully addressed all the points raised and made the necessary revisions to ensure

the manuscript meets the journal’s standards. Thank you for your time and effort in

reviewing our work.

Attachment Submitted filename: Point by point response.pdf

# Peer review: Decision Letter 1

**DOI:** 10.1371/journal.pone.0354013.r003

**Authors:** Saki Raheem

16 Feb 2026

PONE-D-25-53988R1

Integrated Computational and Antimicrobial Study of Efflux Pump Inhibitors Targeting NorA in Mastitis-Associated Staphylococcus aureus

PLOS One

Dear Dr. Kifle,

Thank you for submitting your manuscript to PLOS ONE. After careful consideration, we feel that it has merit but does not fully meet PLOS ONE’s publication criteria as it currently stands. Therefore, we invite you to submit a revised version of the manuscript that addresses the points raised during the review process.

Please submit your revised manuscript by Apr 02 2026 11:59PM. If you will need more time than this to complete your revisions, please reply to this message or contact the journal office at plosone@plos.org. When you're ready to submit your revision, log on to https://www.editorialmanager.com/pone/ and select the 'Submissions Needing Revision' folder to locate your manuscript file.

Please include the following items when submitting your revised manuscript:

- A letter that responds to each point raised by the academic editor and reviewer(s). You should upload this letter as a separate file labeled 'Response to Reviewers'.
- A marked-up copy of your manuscript that highlights changes made to the original version. You should upload this as a separate file labeled 'Revised Manuscript with Track Changes'.
- An unmarked version of your revised paper without tracked changes. You should upload this as a separate file labeled 'Manuscript'.

If you would like to make changes to your financial disclosure, please include your updated statement in your cover letter. Guidelines for resubmitting your figure files are available below the reviewer comments at the end of this letter.

If applicable, we recommend that you deposit your laboratory protocols in protocols.io to enhance the reproducibility of your results. Protocols.io assigns your protocol its own identifier (DOI) so that it can be cited independently in the future. For instructions see: https://journals.plos.org/plosone/s/submission-guidelines#loc-laboratory-protocols. Additionally, PLOS ONE offers an option for publishing peer-reviewed Lab Protocol articles, which describe protocols hosted on protocols.io. Read more information on sharing protocols at https://plos.org/protocols?utm_medium=editorial-email&utm_source=authorletters&utm_campaign=protocols.

We look forward to receiving your revised manuscript.

Kind regards,

Saki Raheem, PhD

Academic Editor

PLOS One

Reviewers' comments:

Reviewer's Responses to Questions

**Comments to the Author**

1. If the authors have adequately addressed your comments raised in a previous round of review and you feel that this manuscript is now acceptable for publication, you may indicate that here to bypass the “Comments to the Author” section, enter your conflict of interest statement in the “Confidential to Editor” section, and submit your "Accept" recommendation.

Reviewer #1: All comments have been addressed

Reviewer #2: (No Response)

Reviewer #3: (No Response)

**********

2. Is the manuscript technically sound, and do the data support the conclusions?

The manuscript must describe a technically sound piece of scientific research with data that supports the conclusions. Experiments must have been conducted rigorously, with appropriate controls, replication, and sample sizes. The conclusions must be drawn appropriately based on the data presented.

Reviewer #1: Yes

Reviewer #2: Partly

Reviewer #3: Partly

**********

3. Has the statistical analysis been performed appropriately and rigorously?

Reviewer #1: Yes

Reviewer #2: I Don't Know

Reviewer #3: No

**********

4. Have the authors made all data underlying the findings in their manuscript fully available?

The PLOS Data policy requires authors to make all data underlying the findings described in their manuscript fully available without restriction, with rare exception (please refer to the Data Availability Statement in the manuscript PDF file). The data should be provided as part of the manuscript or its supporting information, or deposited to a public repository. For example, in addition to summary statistics, the data points behind means, medians and variance measures should be available. If there are restrictions on publicly sharing data—e.g. participant privacy or use of data from a third party—those must be specified.

Reviewer #1: Yes

Reviewer #2: Yes

Reviewer #3: Yes

**********

5. Is the manuscript presented in an intelligible fashion and written in standard English?

PLOS ONE does not copyedit accepted manuscripts, so the language in submitted articles must be clear, correct, and unambiguous. Any typographical or grammatical errors should be corrected at revision, so please note any specific errors here.

Reviewer #1: Yes

Reviewer #2: Yes

Reviewer #3: No

**********

6. Review Comments to the Author

Please use the space provided to explain your answers to the questions above. You may also include additional comments for the author, including concerns about dual publication, research ethics, or publication ethics. (Please upload your review as an attachment if it exceeds 20,000 characters)

Reviewer #1: Dear Authors

Thanks for correcting the manuscript.

Reviewer #2: Thank you for giving me an opportunity to review the revised manuscript entitled “Integrated Computational and Antimicrobial Study of Efflux Pump Inhibitors Targeting NorA in Mastitis-Associated Staphylococcus aureus” submitted to Plos one.

1. The authors are encouraged to target norB, norC, and mepA, which are also key contributors to antimicrobial resistance in Staphylococcus aureus. Their inclusion in the results would likely enhance the scientific value of the study.

2. The authors should carry out in vitro assays to validate the inhibitory effects of 3-(6-methyl-3-pyridyl)-1,5-diphenyl-2-pyrazoline and octadecanoic acid on bacterial efflux pumps.

https://doi.org/10.3390/ph18030323

https://doi.org/10.1080/07391102.2021.1976277

https://doi.org/10.1016/j.prenap.2024.100070

https://doi.org/10.1007/s00284-024-03836-0

https://doi.org/10.3389/fmicb.2018.02990

I recommend acceptance of the manuscript after major Revision.

In light of my overall critical evolution, I found that your manuscript is not suitable for publication in its current form.

Reviewer #3: 1. The manuscript still requires substantial professional English language editing. Numerous grammatical errors, awkward phrases, and overly long or broken sentences remain throughout the text, despite the authors’ claim of thorough revision. A careful, line-by-line edit by a native English speaker or professional editing service is necessary to improve clarity and readability.

2. The reference list is incomplete and inconsistently formatted. Several entries lack essential elements such as journal names, full author details, and page numbers. The entire reference section must be reconstructed according to a consistent standard style, following the journal guidelines, ensuring that all citations are complete and properly punctuated.

3. There is inconsistency between the response letter and the revised manuscript. Some claimed revisions are not fully reflected in the clean version of the manuscript, and discrepancies remain between the tracked-changes version and the final submitted file. The authors should carefully reconcile both versions to ensure accuracy and completeness of all revisions.

4. The concentration of methanol used for extraction is missing in the Methods section. Although 80% methanol is mentioned in the response letter, this detail does not appear in the manuscript. The exact concentration must be clearly stated to ensure reproducibility.

5. Compound nomenclature is inconsistent across the manuscript. In particular, the phthalate compound is referred to by different names in the text and tables. A single, consistent chemical name should be used throughout all sections, tables, and figures.

6. Chemical formulas are not consistently formatted with proper subscripts and superscripts. A final technical check is required to ensure that all molecular formulas are correctly formatted in the text, tables, and figures.

7. The preparation of the extract solution used for bioassays is insufficiently described. The manuscript should clearly detail how the extract was dissolved, diluted, and prepared prior to antimicrobial testing to ensure methodological transparency.

8. Minimum inhibitory concentration (MIC) values are not reported. These values should be provided to properly support and quantify the antibacterial activity claims. The current disk diffusion assay is limited.

10. A full description of bacterial features should be reported, including the source/origin and resistance profile. Additionally, the reference strains should be included to ensure methodological reproducibility.

11. A validation of computational results would add a significant value to the study.

12. The justification for using GC–MS analysis on a methanol extract is unclear. Since GC–MS is typically suited for volatile and semi-volatile compounds, the authors should justify its appropriateness for this extract or clarify any derivatization or methodological considerations.

13. The manuscript is excessively long, particularly in the Results and Discussion sections. Redundant explanations and overly detailed analysis should be reduced to improve focus, clarity, and overall readability.

**********

7. PLOS authors have the option to publish the peer review history of their article (what does this mean?). If published, this will include your full peer review and any attached files.

If you choose “no”, your identity will remain anonymous but your review may still be made public.

**Do you want your identity to be public for this peer review?**  For information about this choice, including consent withdrawal, please see our Privacy Policy.

Reviewer #1: **Yes:** Michel Hanania

Reviewer #2: No

Reviewer #3: **Yes:** Armel Jackson Seukep

**********

[NOTE: If reviewer comments were submitted as an attachment file, they will be attached to this email and accessible via the submission site. Please log into your account, locate the manuscript record, and check for the action link "View Attachments". If this link does not appear, there are no attachment files.]

To ensure your figures meet our technical requirements, please review our figure guidelines: https://journals.plos.org/plosone/s/figures

You may also use PLOS’s free figure tool, NAAS, to help you prepare publication quality figures: https://journals.plos.org/plosone/s/figures#loc-tools-for-figure-preparation.

NAAS will assess whether your figures meet our technical requirements by comparing each figure against our figure specifications.

# Author comment: Author response to Decision Letter 2

**DOI:** 10.1371/journal.pone.0354013.r004

19 Apr 2026

RESPONSE TO REVIEWERS COMMENTS

Reviewer #1

Comment: “Dear Authors

Thanks for correcting the manuscript.”

Authors Response: We are grateful for your response respected reviewer.

Reviewer #2

Comment 1: The authors are encouraged to target norB, norC, and mepA, which are also key contributors to antimicrobial resistance in Staphylococcus aureus. Their inclusion in the results would likely enhance the scientific value of the study.

Authors Response: Thanks a lot dear reviewer for suggestion. Now we have also added NorB, NorC, and mepA, targets in our manuscript as per your suggestion.

Comment 2: The authors should carry out in vitro assays to validate the inhibitory effects of 3-(6-methyl-3-pyridyl)-1,5-diphenyl-2-pyrazoline and octadecanoic acid on bacterial efflux pumps.

https://doi.org/10.3390/ph18030323

https://doi.org/10.1080/07391102.2021.1976277

https://doi.org/10.1016/j.prenap.2024.100070

https://doi.org/10.1007/s00284-024-03836-0

https://doi.org/10.3389/fmicb.2018.02990

Authors Response: We sincerely appreciate the reviewer’s suggestion to validate the inhibitory effects of 3-(6‑methyl‑3‑pyridyl)‑1,5‑diphenyl‑2‑pyrazoline and octadecanoic acid on bacterial efflux pumps through in vitro assays. In the revised manuscript, we have incorporated a comprehensive experimental evaluation that includes both well diffusion antibacterial assays and Minimum Inhibitory Concentration (MIC) determinations. Additionally, we have discussed these in vitro results in the context of efflux pump inhibition and supported our interpretations with relevant literature.

Reviewer #3

Comment 1: The manuscript still requires substantial professional English language editing. Numerous grammatical errors, awkward phrases, and overly long or broken sentences remain throughout the text, despite the authors’ claim of thorough revision. A careful, line-by-line edit by a native English speaker or professional editing service is necessary to improve clarity and readability.

Authors Response: Thank you for this important comment. We performed a comprehensive, line-by-line English language edit across the entire manuscript to improve clarity, grammar, and readability. Specifically, we corrected grammatical and typographical errors, rephrased awkward constructions, reduced redundancy, and split overly long sentences. We also standardized technical terminology and abbreviations throughout the text to ensure consistency. These revisions have been implemented throughout the manuscript.

Comment 2: The reference list is incomplete and inconsistently formatted. Several entries lack essential elements such as journal names, full author details, and page numbers. The entire reference section must be reconstructed according to a consistent standard style, following the journal guidelines, ensuring that all citations are complete and properly punctuated.

Authors Response: Thanks a lot dear reviewer for pointing out. We have now revised the whole manuscript as per your suggestions,

Comment 3: There is inconsistency between the response letter and the revised manuscript. Some claimed revisions are not fully reflected in the clean version of the manuscript, and discrepancies remain between the tracked-changes version and the final submitted file. The authors should carefully reconcile both versions to ensure accuracy and completeness of all revisions.

Authors Response: We thank the reviewer for pointing this out. We have carefully double-checked and reconciled the tracked-changes version with the clean version of the manuscript to ensure that all revisions described in the response letter are accurately reflected. All updates, including changes to compound nomenclature, GC–MS justification, extract preparation, antibacterial assays, MIC values, and formatting corrections, have now been thoroughly incorporated to ensure consistency and completeness.

Comment 4: The concentration of methanol used for extraction is missing in the Methods section. Although 80% methanol is mentioned in the response letter, this detail does not appear in the manuscript. The exact concentration must be clearly stated to ensure reproducibility.

Authors Response: Thank you dear reviwer for pointing this out. The Methods section has been revised to explicitly state the solvent composition used for extraction. Specifically, the extract was prepared using 80% (v/v) methanol (methanol:water, 80:20) (Section 2.1).

Comment 5: Compound nomenclature is inconsistent across the manuscript. In particular, the phthalate compound is referred to by different names in the text and tables. A single, consistent chemical name should be used throughout all sections, tables, and figures.

Authors Response: We thank the reviewer for pointing out the inconsistencies in compound nomenclature throughout the manuscript. We carefully reviewed the entire text, tables, figures, and supplementary materials, and standardized the naming of all chemical compounds to ensure clarity and reproducibility.

Comment 6: Chemical formulas are not consistently formatted with proper subscripts and superscripts. A final technical check is required to ensure that all molecular formulas are correctly formatted in the text, tables, and figures.

Authors Response: We thank the reviewer for highlighting the inconsistency in the formatting of chemical formulas throughout the manuscript. We have carefully reviewed all text, tables, figures, and supplementary materials, and corrected the formatting to ensure that all molecular formulas display proper subscripts and superscripts according to standard chemical notation.

Comment 7: The preparation of the extract solution used for bioassays is insufficiently described. The manuscript should clearly detail how the extract was dissolved, diluted, and prepared prior to antimicrobial testing to ensure methodological transparency.

Authors Response: We thank the reviewer for pointing out the need for greater detail regarding the preparation of the Pisolithus arhizus extract solution for bioassays. In the revised manuscript, we have clarified the procedure to ensure methodological transparency and reproducibility. The Materials and Methods section now specifies how the methanolic extract was dissolved, diluted, and prepared prior to antimicrobial testing.

Comment 8: Minimum inhibitory concentration (MIC) values are not reported. These values should be provided to properly support and quantify the antibacterial activity claims. The current disk diffusion assay is limited.

Authors Response: We thank the reviewer for emphasizing the importance of reporting Minimum Inhibitory Concentration (MIC) values to support and quantify the antibacterial activity. In the revised manuscript, we have now included MIC determinations for all tested samples.

Comment 9: A full description of bacterial features should be reported, including the source/origin and resistance profile. Additionally, the reference strains should be included to ensure methodological reproducibility.

Authors Response: We thank the reviewer for pointing out; The need for a full description of the bacterial strain, including its source, reference number, and resistance profile, to ensure reproducibility of the antibacterial assays. In the revised manuscript, we have updated the Materials and Methods section to provide this information in detail.

Comment 10: A validation of computational results would add a significant value to the study.

Authors Response: We thank the reviewer for highlighting the importance of validating the computational predictions. In the revised manuscript, we have incorporated in vitro antibacterial and MIC assays to support the in silico findings. Specifically, 3-(6-methyl-3-pyridyl)-1,5-diphenyl-2-pyrazoline (pyrazoline) and octadecanoic acid, which were predicted via molecular docking and MM-GBSA analyses to interact strongly with Staphylococcus aureus efflux pumps, were tested for antibacterial activity using well diffusion and Minimum Inhibitory Concentration (MIC) assays against S. aureus (ATCC 23235).

Comment 11: The justification for using GC–MS analysis on a methanol extract is unclear. Since GC–MS is typically suited for volatile and semi-volatile compounds, the authors should justify its appropriateness for this extract or clarify any derivatization or methodological considerations.

Authors Response: We thank the reviewer for the comment. We have updated the manuscript to clarify that GC–MS was used because the Pisolithus arhizus methanolic extract contains volatile and semi-volatile metabolites (fatty acids, phthalates, xylene derivatives, and pyrazoline derivatives) suitable for direct GC–MS analysis. No derivatization was required, as the compounds were sufficiently volatile and thermally stable.

Comment 12: The manuscript is excessively long, particularly in the Results and Discussion sections. Redundant explanations and overly detailed analysis should be reduced to improve focus, clarity, and overall readability.

Authors Response: Thanks a lot dear reviewer for your comment. Now we have updated the manuscript.

Comments to editor:

Dear Editor’s,

We have carefully addressed all the comments and suggestions provided. The revisions have been made accordingly, with changes highlighted in red text and grammatical improvements marked in blue text. We believe these revisions have strengthened the manuscript, and we hope it now meets the journal's standards. Thank you for your time and consideration.

Attachment Submitted filename: Point by point response final.docx

# Peer review: Decision Letter 2

**DOI:** 10.1371/journal.pone.0354013.r005

**Authors:** Saki Raheem

25 May 2026

PONE-D-25-53988R2

Computational and Experimental Evaluation of Pisolithus arhizus Metabolites on Major Mastitis-Associated S. aureus Efflux Pumps

PLOS One

Dear Dr. Kifle,

Thank you for submitting your manuscript to PLOS ONE. After careful consideration, we feel that it has merit but does not fully meet PLOS ONE’s publication criteria as it currently stands. Therefore, we invite you to submit a revised version of the manuscript that addresses the points raised during the review process.

Please submit your revised manuscript by Jul 09 2026 11:59PM. If you will need more time than this to complete your revisions, please reply to this message or contact the journal office at plosone@plos.org. When you're ready to submit your revision, log on to https://www.editorialmanager.com/pone/ and select the 'Submissions Needing Revision' folder to locate your manuscript file.

Please include the following items when submitting your revised manuscript:

- A letter that responds to each point raised by the academic editor and reviewer(s). You should upload this letter as a separate file labeled 'Response to Reviewers'.
- A marked-up copy of your manuscript that highlights changes made to the original version. You should upload this as a separate file labeled 'Revised Manuscript with Track Changes'.
- An unmarked version of your revised paper without tracked changes. You should upload this as a separate file labeled 'Manuscript'.

If you would like to make changes to your financial disclosure, please include your updated statement in your cover letter. Guidelines for resubmitting your figure files are available below the reviewer comments at the end of this letter.

If applicable, we recommend that you deposit your laboratory protocols in protocols.io to enhance the reproducibility of your results. Protocols.io assigns your protocol its own identifier (DOI) so that it can be cited independently in the future. For instructions see: https://journals.plos.org/plosone/s/submission-guidelines#loc-laboratory-protocols. Additionally, PLOS ONE offers an option for publishing peer-reviewed Lab Protocol articles, which describe protocols hosted on protocols.io. Read more information on sharing protocols at https://plos.org/protocols?utm_medium=editorial-email&utm_source=authorletters&utm_campaign=protocols.

As the corresponding author, your ORCID iD is verified in the submission system and will appear in the published article. PLOS supports the use of ORCID, and we encourage all coauthors to register for an ORCID iD and use it as well. Please encourage your coauthors to verify their ORCID iD within the submission system before final acceptance, as unverified ORCID iDs will not appear in the published article. *Only*  the individual author can complete the verification step; PLOS staff *cannot*  verify ORCID iDs on behalf of authors.

We look forward to receiving your revised manuscript.

Kind regards,

Saki Raheem, PhD

Academic Editor

PLOS One

Journal Requirements:

If the reviewer comments include a recommendation to cite specific previously published works, please review and evaluate these publications to determine whether they are relevant and should be cited. There is no requirement to cite these works unless the editor has indicated otherwise.

Please review your reference list to ensure that it is complete and correct. If you have cited papers that have been retracted, please include the rationale for doing so in the manuscript text, or remove these references and replace them with relevant current references. Any changes to the reference list should be mentioned in the rebuttal letter that accompanies your revised manuscript. If you need to cite a retracted article, indicate the article’s retracted status in the References list and also include a citation and full reference for the retraction notice.

Reviewers' comments:

Reviewer's Responses to Questions

**Comments to the Author**

1. If the authors have adequately addressed your comments raised in a previous round of review and you feel that this manuscript is now acceptable for publication, you may indicate that here to bypass the “Comments to the Author” section, enter your conflict of interest statement in the “Confidential to Editor” section, and submit your "Accept" recommendation.

Reviewer #3: (No Response)

**********

2. Is the manuscript technically sound, and do the data support the conclusions?

The manuscript must describe a technically sound piece of scientific research with data that supports the conclusions. Experiments must have been conducted rigorously, with appropriate controls, replication, and sample sizes. The conclusions must be drawn appropriately based on the data presented.

Reviewer #3: Partly

**********

3. Has the statistical analysis been performed appropriately and rigorously?

Reviewer #3: No

**********

4. Have the authors made all data underlying the findings in their manuscript fully available?

The PLOS Data policy requires authors to make all data underlying the findings described in their manuscript fully available without restriction, with rare exception (please refer to the Data Availability Statement in the manuscript PDF file). The data should be provided as part of the manuscript or its supporting information, or deposited to a public repository. For example, in addition to summary statistics, the data points behind means, medians and variance measures should be available. If there are restrictions on publicly sharing data—e.g. participant privacy or use of data from a third party—those must be specified.

Reviewer #3: Yes

**********

5. Is the manuscript presented in an intelligible fashion and written in standard English?

PLOS ONE does not copyedit accepted manuscripts, so the language in submitted articles must be clear, correct, and unambiguous. Any typographical or grammatical errors should be corrected at revision, so please note any specific errors here.

Reviewer #3: Yes

**********

6. Review Comments to the Author

Please use the space provided to explain your answers to the questions above. You may also include additional comments for the author, including concerns about dual publication, research ethics, or publication ethics. (Please upload your review as an attachment if it exceeds 20,000 characters)

Reviewer #3: The manuscript has improved substantially compared with the previous version, but important issues remain regarding:

- Overinterpretation of efflux pump inhibition,

- persistent language problems,

- incomplete integration of NorC analyses,

- and excessive speculative discussion.

A further moderate revision focused on scientific rigor, consistency, and language quality is still recommended. More specifically:

1. The manuscript still overstates the evidence for efflux pump inhibition. The additional experiments presented only demonstrate antibacterial activity and do not directly validate efflux pump inhibition. Statements linking antibacterial effects to efflux inhibition therefore remain speculative and should be moderated throughout the manuscript unless supported by dedicated efflux-related assays such as ethidium bromide accumulation assay, efflux inhibition assay, synergism assay with antibiotics, or transporter-specific validation

2. Despite the authors’ claim of extensive language revision, numerous grammatical, stylistic, and sentence-construction issues persist across the manuscript. Several expressions remain awkward or scientifically imprecise, indicating that the manuscript still requires thorough professional English editing before publication.

3. There is also inconsistency regarding the inclusion of the NorC target in the computational analyses. While the authors state that NorC was incorporated comprehensively, several downstream analyses, figures, and tables appear to include only NorA, NorB, and MepA. This discrepancy should be clarified and corrected for consistency.

4. The revised manuscript remains excessively long and repetitive, particularly in the Results and Discussion sections. Similar explanations of molecular interactions and antibacterial relevance are repeated multiple times, and the text would benefit from substantial condensation to improve clarity and readability.

5. Although the authors acknowledged that the pyrazoline compound identification remains tentative based on GC–MS library matching, the manuscript still discusses the compound in several sections as though it were definitively identified and biologically confirmed. The tentative nature of the identification should be consistently reflected throughout the text.

6. Some methodological and statistical details remain insufficiently reported or inconsistently formatted. These include unclear presentation of CFU values, limited reporting of statistical analyses and p-values, and inadequate distinction between biological and technical replicates.

7. Finally, several scientific interpretations remain overly speculative and extend beyond the experimental evidence provided. Claims related to membrane targeting, wound healing relevance, therapeutic applications, and broad-spectrum efflux inhibition should be tempered to better align with the data generated in the study.

**********

7. PLOS authors have the option to publish the peer review history of their article (what does this mean?). If published, this will include your full peer review and any attached files.

If you choose “no”, your identity will remain anonymous but your review may still be made public.

**Do you want your identity to be public for this peer review?**  For information about this choice, including consent withdrawal, please see our Privacy Policy.

Reviewer #3: **Yes:** Armel Jackson Seukep, PhD

**********

[NOTE: If reviewer comments were submitted as an attachment file, they will be attached to this email and accessible via the submission site. Please log into your account, locate the manuscript record, and check for the action link "View Attachments". If this link does not appear, there are no attachment files.]

To ensure your figures meet our technical requirements, please review our figure guidelines: https://journals.plos.org/plosone/s/figures

You may also use PLOS’s free figure tool, NAAS, to help you prepare publication quality figures: https://journals.plos.org/plosone/s/figures#loc-tools-for-figure-preparation.

NAAS will assess whether your figures meet our technical requirements by comparing each figure against our figure specifications.

# Author comment: Author response to Decision Letter 3

**DOI:** 10.1371/journal.pone.0354013.r006

15 Jun 2026

Dear Reviewer, thank you for your constructive and highly valuable comments. We have carefully gone through your comments and tried to address them point by point. The point-by-point responses to each and every comment letter is attached to this platform along with the revised version of the manuscript.

Thanks and Regards.

Dawit Kifle (PhD)

corresponding Author.

Attachment Submitted filename: point to point responses R4.pdf

# Editor report: Decision Letter 3

**DOI:** 10.1371/journal.pone.0354013.r007

**Authors:** Saki Raheem

2 Jul 2026

Computational and Experimental Evaluation of Pisolithus arhizus Metabolites Targeting Major Efflux Pumps of Mastitis-Associated Staphylococcus aureus

PONE-D-25-53988R3

Dear Dr. Kifle,

We’re pleased to inform you that your manuscript has been judged scientifically suitable for publication and will be formally accepted for publication once it meets all outstanding technical requirements.

Within one week, you’ll receive an e-mail detailing the required amendments. When these have been addressed, you’ll receive a formal acceptance letter and your manuscript will be scheduled for publication.

An invoice will be generated when your article is formally accepted. Please note, if your institution has a publishing partnership with PLOS and your article meets the relevant criteria, all or part of your publication costs will be covered. Please make sure your user information is up-to-date by logging into Editorial Manager at Editorial Manager® and clicking the ‘Update My Information' link at the top of the page. For questions related to billing, please contact billing support.

If your institution or institutions have a press office, please notify them about your upcoming paper to help maximize its impact. If they’ll be preparing press materials, please inform our press team as soon as possible -- no later than 48 hours after receiving the formal acceptance. Your manuscript will remain under strict press embargo until 2 pm Eastern Time on the date of publication. For more information, please contact onepress@plos.org.

Kind regards,

Saki Raheem, PhD

Academic Editor

PLOS One

# Editor report: Acceptance letter

**DOI:** 10.1371/journal.pone.0354013.r008

**Authors:** Saki Raheem

PONE-D-25-53988R3

PLOS One

Dear Dr. Kifle,

I'm pleased to inform you that your manuscript has been deemed suitable for publication in PLOS One. Congratulations! Your manuscript is now being handed over to our production team.

At this stage, our production department will prepare your paper for publication. This includes ensuring the following:

* All references, tables, and figures are properly cited

* All relevant supporting information is included in the manuscript submission,

* There are no issues that prevent the paper from being properly typeset

You will receive further instructions from the production team, including instructions on how to review your proof when it is ready. Please keep in mind that we are working through a large volume of accepted articles, so please give us a few days to review your paper and let you know the next and final steps.

Lastly, if your institution or institutions have a press office, please let them know about your upcoming paper now to help maximize its impact. If they'll be preparing press materials, please inform our press team within the next 48 hours. Your manuscript will remain under strict press embargo until 2 pm Eastern Time on the date of publication. For more information, please contact onepress@plos.org.

You will receive an invoice from PLOS for your publication fee after your manuscript has reached the completed accept phase. If you receive an email requesting payment before acceptance or for any other service, this may be a phishing scheme. Learn how to identify phishing emails and protect your accounts at https://explore.plos.org/phishing.

If we can help with anything else, please email us at customercare@plos.org.

Thank you for submitting your work to PLOS ONE and supporting open access.

Kind regards,

PLOS ONE Editorial Office Staff

on behalf of

Dr. Saki Raheem

Academic Editor

PLOS One
